# ROS-responsive hydrogels with spatiotemporally sequential delivery of antibacterial and anti-inflammatory drugs for the repair of MRSA-infected wounds

**DOI:** 10.1093/rb/rbad110

**Published:** 2023-12-09

**Authors:** Bowen Qiao, Jiaxin Wang, Lipeng Qiao, Aziz Maleki, Yongping Liang, Baolin Guo

**Affiliations:** Key Laboratory of Shaanxi Province for Craniofacial Precision Medicine Research, College of Stomatology, Xi’an Jiaotong University, Xi’an 710049, China; State Key Laboratory for Mechanical Behavior of Materials, Frontier Institute of Science and Technology, Xi’an Jiaotong University, Xi’an 710049, China; State Key Laboratory for Mechanical Behavior of Materials, Frontier Institute of Science and Technology, Xi’an Jiaotong University, Xi’an 710049, China; State Key Laboratory for Mechanical Behavior of Materials, Frontier Institute of Science and Technology, Xi’an Jiaotong University, Xi’an 710049, China; Zanjan Pharmaceutical Nanotechnology Research Center (ZPNRC), Zanjan 45139-56184, Iran; State Key Laboratory for Mechanical Behavior of Materials, Frontier Institute of Science and Technology, Xi’an Jiaotong University, Xi’an 710049, China; Key Laboratory of Shaanxi Province for Craniofacial Precision Medicine Research, College of Stomatology, Xi’an Jiaotong University, Xi’an 710049, China; State Key Laboratory for Mechanical Behavior of Materials, Frontier Institute of Science and Technology, Xi’an Jiaotong University, Xi’an 710049, China; Department of Orthopaedics, The First Affiliated Hospital of Xi’an Jiaotong University, Xi’an 710061, People’s Republic of China

**Keywords:** MRSA-infected wounds, spatiotemporally sequential delivery, antibacterial, anti-inflammatory

## Abstract

For the treatment of MRSA-infected wounds, the spatiotemporally sequential delivery of antibacterial and anti-inflammatory drugs is a promising strategy. In this study, ROS-responsive HA-PBA/PVA (HPA) hydrogel was prepared by phenylborate ester bond cross-linking between hyaluronic acid-grafted 3-amino phenylboronic acid (HA-PBA) and polyvinyl alcohol (PVA) to achieve spatiotemporally controlled release of two kinds of drug to treat MRSA-infected wound. The hydrophilic antibiotic moxifloxacin (M) was directly loaded in the hydrogel. And hydrophobic curcumin (Cur) with anti-inflammatory function was first mixed with Pluronic F127 (PF) to form Cur-encapsulated PF micelles (Cur-PF), and then loaded into the HPA hydrogel. Due to the different hydrophilic and hydrophobic nature of moxifloxacin and Cur and their different existing forms in the HPA hydrogel, the final HPA/M&Cur-PF hydrogel can achieve different spatiotemporally sequential delivery of the two drugs. In addition, the swelling, degradation, self-healing, antibacterial, anti-inflammatory, antioxidant property, and biocompatibility of hydrogels were tested. Finally, in the MRSA-infected mouse skin wound, the hydrogel-treated group showed faster wound closure, less inflammation and more collagen deposition. Immunofluorescence experiments further confirmed that the hydrogel promoted better repair by reducing inflammation (TNF-α) and promoting vascular (VEGF) regeneration. In conclusion, this HPA/M&Cur-PF hydrogel that can spatiotemporally sequential deliver antibacterial and anti-inflammatory drugs showed great potential for the repair of MRSA-infected skin wounds.

## Graphical Abstract

**Figure:**
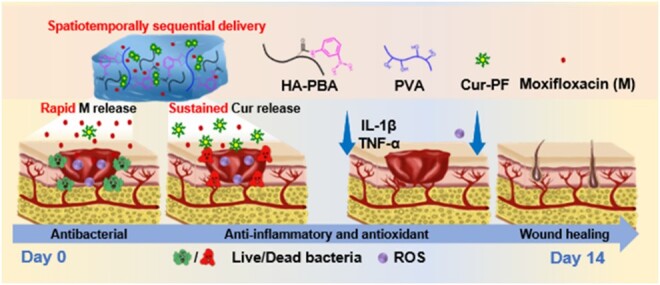

## Introduction

The skin separates the internal environment of the human body from the external environment and plays a crucial role in the human body [1, 2]. However, skin is very vulnerable to injury, which includes external factors (surgery, pressure, burns and cuts, etc.) and pathological factors (diabetes or vascular disease, etc.) [3]. These injuries can cause gaps in the protective barrier of the skin, allowing pathogens such as bacteria to attack the human body through gaps. Research showed that methicillin-resistant *Staphylococcus aureus* (MRSA) can exist in 7–30% of wounds [4], and MRSA may spread into the blood, even endanger life. Because MRSA is an antibiotic-resistant pathogen that can cause multiple serious infections, research by the World Health Organization have shown that the mortality rate of MRSA-infected patients is 64% higher than that of other infected patients [5]. Therefore, effective treatment strategies for MRSA infection are particularly important for people’s lives and health.

At present, there are many strategies for the treatment of MRSA-infected wounds, such as antibiotics, bacteriophages and nanomedicine platforms [6, 7]. Among numerous treatment methods, the use of antibiotics that can kill drug-resistant bacteria can directly and effectively treat MRSA infection, but the unreasonable use of drug doses gradually reduces the therapeutic effect of antibiotics, and even forms the antibiotic resistance [8, 9]. Therefore, designing and developing better biocarrier to control antibiotics release is a mainstream direction for the treatment of MRSA infection [10–12]. Among the current medical wound dressings (gauze, adhesive bandage, foam and hydrogel, etc.) [13–15], hydrogel is a good platform for antibiotic controlled release [16]. At the same time, hydrogel offers the advantages that other materials do not have, such as moisturizing, self-healing, and on-demand functional designs [17, 18]. Therefore, it is of great practical significance to develop an antibacterial hydrogel-based antibiotic delivery system for MRSA-infected wounds.

After the formation of the wound, the locally recruited inflammatory cells immediately migrate to the wound site, making the wound repair enter the inflammatory phase [19, 20]. However, excessive oxidative stress at the wound site can produce excessive ROS [21], which in turn triggers chronic inflammation [22], creating a vicious cycle. On the other hand, the colonization of MRSA in the wound bed undoubtedly leads to severe infection [23], exacerbating the inflammatory response and causing tissue damage [24]. Infection also weakens immune cells, making it difficult to reverse pathological changes, prolonging the inflammatory phase and hindering further wound repair [25, 26]. Therefore, in order to treat infected wounds, it is necessary to remove bacteria [27, 28], reduce ROS and inflammation [29–31], so as to restore balance in the microenvironment [32, 33] of the wound site and facilitate orderly subsequent repair.

There have been studies on hydrogels for the treatment of MRSA infection from the perspective of antibacterial, anti-inflammatory and antioxidant, but the load of drugs is mostly reflected in the controlled release of a single drug. For example, due to the presence of carboxyl groups in pectin and gelatin, curcumin (Cur)-loaded photocrosslinked hydrogels composed of methacrylated gelatin and methacrylated pectin can release more Cur under alkaline conditions, showing great advantages for the treatment of infected wounds [34]. Singh *et al.* prepared a hydrogel system by chitosan and poly(N-isopropylacrylamide-co-methacrylic acid) (PNIPAM-co-MAA) microgels. Due to the temperature-responsiveness of PNIPAM and the pH-responsiveness of the carboxylic acid groups in MAA, the release of moxifloxacin in the hydrogel can achieve dual-responsive control of temperature and pH [35]. The above studies have shown that a single controlled release of a drug can only achieve a single purpose, which cannot meet the multiple needs of infected wounds for antibacterial and anti-inflammatory etc., and also cannot meet the spatiotemporally sequential delivery of multiple needs. Therefore, design of hydrogel dressings that can deliver different drugs at different times to treat MRSA-infected wounds is a promising strategy.

Moxifloxacin is a broad-spectrum fluoroquinolone antibiotic [36], which is an appropriate choice for the treatment of skin bacterial infections. Curcumin is a kind of diketone polyphenol compound, which has many functions such as antibacterial, anti-inflammatory, and antioxidant [37–39]. The safety of Cur has been certified by the World Health Organization and U.S. Food and Drug Administration [40]. Besides, the industrial production of Cur is relatively mature, and the product is cheap and economical [41]. Based on the hydrophilicity of moxifloxacin hydrochloride and the hydrophobicity of Cur, they can be designed to exist in two different forms in hydrogels, that is, moxifloxacin hydrochloride can be loaded in the hydrogel by directly mixed with the hydrogel precursor solution. And for Cur, it can be firstly mixed with Pluronic F127 (PF) to form Cur-encapsulated PF micelles (Cur-PF), and then loaded into the hydrogel to achieve sustained release of Cur [42]. So, the release of Cur is characterized by sustained release, while the release of moxifloxacin directly loaded in the hydrogel is characterized by high efficiency and fast release. This is consistent with the treatment characteristics of MRSA-infected wounds, and has not been reported.

In this study, ROS-responsive HPA hydrogel loaded with antibiotic moxifloxacin (M) and anti-inflammatory ingredient Cur-PF was prepared by cross-linking of phenylboronic acid ester between hyaluronic acid-grafted 3-amino phenylboronic acid (HA-PBA) and polyvinyl alcohol (PVA) to treat MRSA-infected wound healing. In this hydrogel, the phenylboronic acid ester bond formed by HA-PBA and PVA has ROS responsiveness, which can realize the responsive release of drugs. Moxifloxacin and Cur exist in different forms in the hydrogel, which makes them spatially different from each other. Besides, the spatial difference between the two drugs results in a difference in their release rate, which further results in a time difference. Therefore, the hydrogel designed above can the hydrogel prepared in this study showed rapidly release of moxifloxacin to endow antibacterial property, and exhibited sustained release of Cur for anti-inflammatory under ROS-responsive conditions. Those differences meet the different needs of MRSA-infected wounds at different treatment periods based on spatiotemporally sequential delivery time-space sequential release of the two drugs. The swelling, degradation, self-healing, biocompatibility, responsive sequential release, antibacterial, anti-inflammatory and antioxidant properties of the hydrogel were tested, and their effectiveness in repairing in full-thickness skin was verified in a MRSA-infected mouse skin wound model. This is the first time to realize the spatiotemporally sequential delivery of antibacterial and anti-inflammatory drugs on hyaluronic acid (HA)-based hydrogel for repairing the MRSA-infected skin wound of mouse.

## Materials and methods

### Materials

Hyaluronic acid (Mn = 800 000 Da), 3-amino phenylboronic acid (PBA), polyvinyl alcohol type 224 (PVA), 1-(3-dimethylaminopropyl)-3-ethyl carbodiimide hydrochloride (EDC), N-hydroxysuccinimide (NHS), and 2,2-diphenyl-1-(2,4,6-trinitrophenyl) hydrazide (DPPH) were purchased from Macklin; Cur, PF, and 2′,7′-dichlorofluorescein diacetate (DCFH-DA) were purchased from Sigma Aldrich. All reagents were used directly without special purification.

### Preparation of hyaluronic acid grafted 3-amino phenylboronic acid (HA-PBA)

HA (1 g, 2.5 mmol) was firstly dissolved in 100 ml of deionized water, then the EDC (575 mg, 3 mmol) and NHS (345 mg, 3 mmol) were added. The pH of the solution was controlled at 5 ∼ 6. After the above mixed solution was stirred for 20 min, PBA (410.65 mg, 3 mmol) was added to the mixture, and 1 M HCl was used to control the pH of the solution at 5 ∼ 6. The reaction lasted overnight at room temperature. Then, the mixture was dialyzed (3500 KD) for 3 days, and freeze-dried.

### Preparation of curcumin encapsulated Pluronic F127 micelles (Cur-PF)

According to the references [43], a one-step solid dispersion method was used to synthesize Cur-PF micelle. The feed ratio of Cur and PF polymer was 2:98.

### Preparation of hydrogels

The synthesized HA-PBA polymer was dissolved in distilled water at a concentration of 3 wt%; PVA was dissolved in distilled water at a concentration of 10 wt%. Then, the HA-PBA and PVA were mixed in a certain volume ratio and vortex rapidly to obtain HPA hydrogels with final HA-PBA concentration of 1.5 wt% and the final PVA concentration of 1, 2 and 3 wt%, and these hydrogels were named as HPA1, HPA2, and HPA3, respectively.

To prepare the hydrogels loaded with Cur and moxifloxacin, the Cur-PF was dissolved in distilled water at a concentration of 30 wt%. Then, Cur-PF and HA-PBA were mixed in advance with a certain volume ratio, and the final concentration of Cur-PF was 5 wt%. The mass content of moxifloxacin and Cur in Cur-PF was the same, and they were premixed into HA-PBA solution. Then, the above mixed solution was mixed with PVA solution, and the hydrogels obtained were named as HPA1/M&Cur-PF, HPA2/M&Cur-PF and HPA3/M&Cur-PF, respectively.

### The characterization of hydrogels

The tests of nuclear magnetic resonance (^1^H-NMR) [44], Fourier transform infrared spectroscopy (FT-IR) [45], field emission scanning electron microscopy (SEM) [46], transmission electron microscope (TEM) [43], swelling [47], degradation [48], self-healing, rheological and mechanical properties [49], DPPH scavenging [50], ROS scavenging [51] and biocompatibility were all carried out according to the literature [52]. And the operational details can be found in SI. All animal experiments were conducted in accordance with the current guidelines for experimental animal care, and were approved by the Professional Committee of Xi’an Jiaotong University.

### *In vitro* drug release assay

The drug release characteristics of HPA/M&Cur-PF hydrogels were tested in PBS or 1 mM H_2_O_2_ for moxifloxacin and Cur. The drug released from the hydrogel was analyzed by UV-Vis spectrophotometer at 420 nm (Cur) and 288.57 nm (Moxifloxacin), respectively [53]. The details can be found in SI.

### Antibacterial property test of the hydrogels

To test the antibacterial properties of the released drugs from the HPA/M&Cur-PF hydrogels, the samples were placed on solid medium (nutrient agar) in contact with the bacteria and the zones of inhibition around each sample were measured to record the antibacterial effect of HPA/M hydrogel loaded with moxifloxacin, and HPA/M&Cur-PF hydrogel loaded with moxifloxacin and Cur [44]. The details can be found in SI.

### Anti-inflammatory experiments of HPA hydrogels

Macrophage polarization was induced by lipopolysaccharide (LPS). Hydrogel leachate was used instead of culture medium, and after incubation for 48 h, total RNA of macrophages was isolated and reverse-transcribed and amplified for further analysis of related gene expression [54].

### Wound healing in an *in vivo* MRSA infection model

To further evaluate the promoting effect of HPA/M&Cur-PF hydrogel on wound healing, a wound healing model of MRSA-infected mouse back skin was established. The details can be found in SI.

### Histological and immunohistochemical evaluation

Collect wound specimens on the Days 3, 7, and 14 after treatment. Then, hematoxylin–eosin (HE) staining was performed to evaluate the epidermal regeneration and inflammation of the wound. Masson staining was used to evaluate collagen deposition in wound beds. On the other hand, immunofluorescence staining was performed using TNF-α and VEGF antibodies, respectively.

### Statistical analysis

All experimental data were statistically analyzed, and the results were expressed as mean ± SD. Statistical differences were determined by one-way ANOVA and a Student *t*-test. In all cases, if *P *<* *0.05, there is a significant difference.

### Ethics approval

All protocols about animal experiments were approved by the animal research committee of Xi’an Jiaotong University (approval number: 2023-1469).

## Result and discussion

### Synthesis of hydrogel

**Figure 1.**
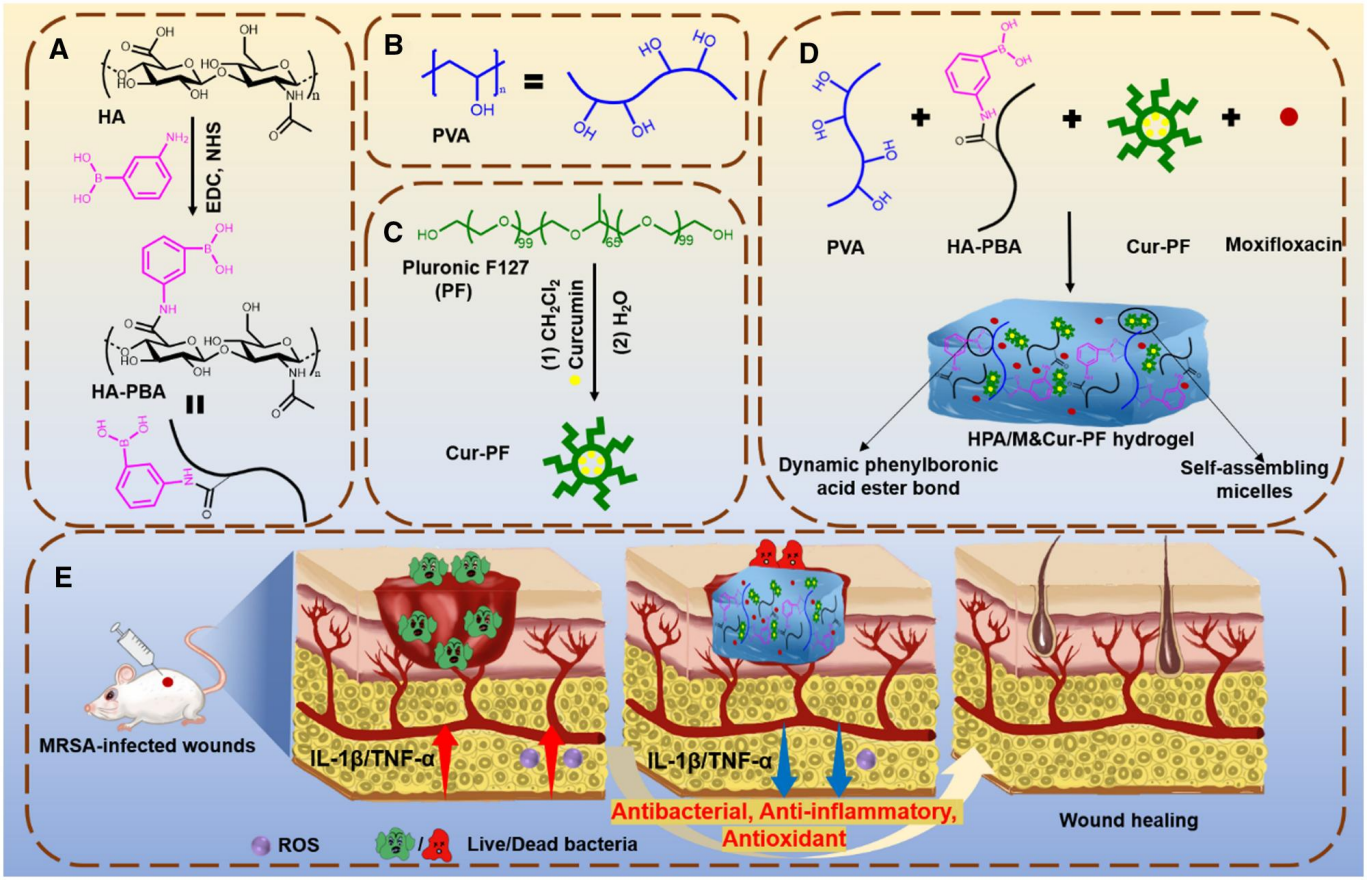
Schematic diagram of preparation and application of HPA/M&Cur-PF hydrogel. (**A**) Hyaluronic acid-grafted 3-amino phenylboronic acid (HA-PBA). (**B**) polyvinyl alcohol (PVA). (**C**) preparation of curcumin-encapsulated pluronic F127 micelles (Cur-PF). (**D**) structure diagram of HPA/M&Cur-PF hydrogel. (**E**) Application of hydrogel in MRSA-infected skin wound healing of mouse.

In this study, based on the dynamic phenylboronic acid ester bond between HA-PBA and PVA, and Cur-PF and antibiotic moxifloxacin (M), a series of hydrogel dressings with good antibacterial, anti-inflammatory and antioxidant effects, and stimulus-responsive drug release in different spatiotemporal sequences were prepared. Figure 1 showed the overall strategy to prepare HPA/M&Cur-PF hydrogels for MRSA-infected skin wounds healing. Firstly, PBA was grafted onto HA through amidation reaction, forming HA-PBA (Figure 1A). Secondly, Cur was encapsulated in PF by taking advantage of the self-assembly characteristics of PF to form Cur-PF (Figure 1B). Figure 1C is the structural diagram of PVA. Figure 1D showed the specific preparation procedure of HPA/M&Cur-PF hydrogel, namely Cur-PF and moxifloxacin were first mixed with HA-PBA precursor solution, and then HA-PBA in the mixed solution formed phenylboronic acid ester dynamic bond through the combination of phenylboronic acid group with the diol group structure on PVA. The hydrogel was named as HA-PBA/PVA1 (HPA1), HA-PBA/PVA2 (HPA2), and HA-PBA/PVA3 (HPA3) according to the final concentration of PVA in the hydrogel varying from 10, 20–30 mg/ml. Figure 1E showed the application of HPA/M&Cur-PF hydrogel in the MRSA-infected skin wound of mice. Based on the response of phenylboronic acid ester dynamic bond to ROS, and the different loading forms of moxifloxacin and Cur in hydrogel, two drugs in the HPA/M&Cur-PF hydrogel achieved stimulus-responsive release in different spatiotemporal sequences. When the HPA/M&Cur-PF hydrogel was applied to the MRSA-infected skin wounds of mice, it can achieve responsive anti-inflammatory and antioxidant property on the basis of rapid antibacterial action, and synergistically promote the wound repair.

**Figure 2.**
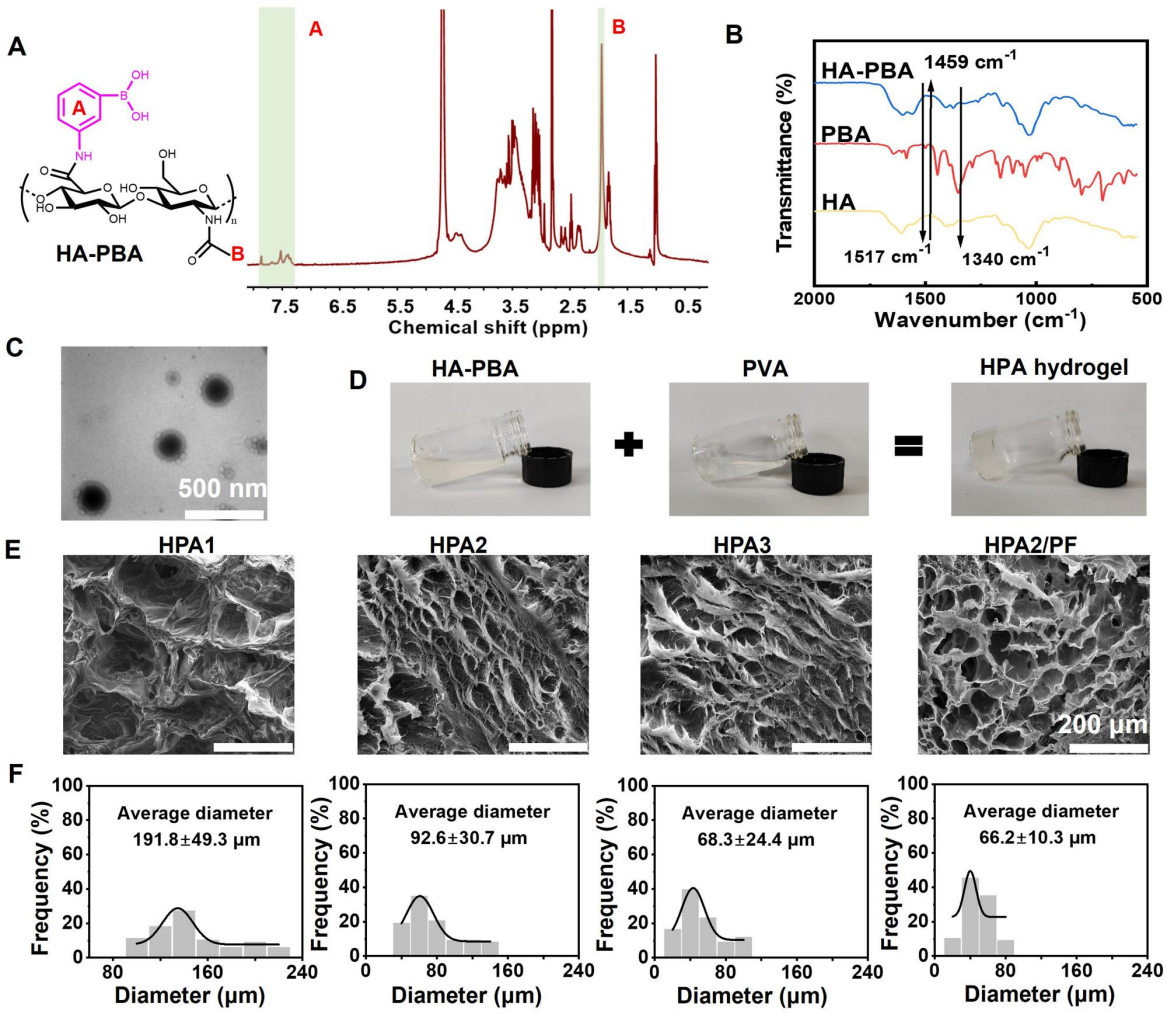
(**A**) ^1^H-NMR spectrum of HA-PBA, A represents the hydrogen on the benzene ring and B represents the hydrogen on the methyl group. (**B**) FT-IR spectra of HA, PBA, and HA-PBA. (**C**) The TEM image of Cur-PF micelle. (**D**) Gelation display of HPA hydrogel. (**E**) HPA1, HPA2, HPA3 and of HPA2/PF hydrogels’ SEM image (scale bar: 200 μm) and (**F**) pore size diameter statistics.

As shown in Figure 2A, the peak of HA-PBA at 7–8 ppm in the ^1^H-NMR spectrum comes from hydrogen on the benzene ring, demonstrating the successful grafting of PBA. The chemical shifts (*δ*, ppm) of the peaks were assigned as below: 7.58 (m, 4H, A), 1.89 (s, 3H, B). It could be seen that phenylboronic acid was successfully grafted. Through integral calculation, the grafting ratio of PBA was 14.2%. Meanwhile, as shown in Figure 2B, the changes in the peaks at 1459 and 1517 cm^−1^ in the FT-IR spectrum came from benzene ring, and the peak at 1340 cm^−1^ is attributed to the stretching vibration of the B–O, once again proving the successful grafting of PBA. The TEM image of Cur-PF in Figure 2C confirmed the formation of Cur-PF micelles with a diameter of around 300 nm, which is consistent with the results of previous studies [43]. As shown in the Supplementary Figure S1, the diameter of Cur-PF micelles was tested by using dynamic light scattering, and the experimental results showed that it is distributed in the range of 220–458 nm, which is consistent with the TEM results. Figure 2D showed the state of HPA hydrogel before and after gelation. Both HA-PBA and PVA are in the liquid state with fluidity. After mixing and shaking them in a certain proportion within 2 min, the gelatinized HPA hydrogel without flowing state can be observed. The test tube inversion method was used to measure the gelation time at constant temperature of 25°C. Supplementary Table S1 showed the average gelation time of the HPA2 hydrogel was 50.4 ± 2.4 s, while the gelation time of the HPA2/PF hydrogel was a little longer, about 69.3 ± 2.1 s, which may be due to the surfactant of PF [55, 56]. Figure 2E showed the SEM images of all hydrogels. With the increase of PVA concentration in HPA1, HPA2 and HPA3 hydrogel, the crosslinking-density of hydrogels was increased, and the pore size also became smaller. When the PF micelles were added, it can be seen that the pore size of HPA2/PF hydrogel was more uniform than that of HPA2, which may be caused by the nature of non-ionic surfactant of PF. Figure 2F showed the statistics of the pore diameter of all hydrogels, which more intuitively showed that with increasing of PVA concentration, the pore size of the hydrogel gradually decreased from 191.8 ± 49.3 µm of HPA1 hydrogel to 92.6 ± 30.7, 68.3 ± 24.4 and 66.2 ± 10.3 µm for HPA2, HPA3 and HPA2/PF hydrogels, respectively. And the pore size of HPA2/PF hydrogel was more uniform compared to HPA2 hydrogel.

### Mechanical properties, swelling, degradation and self-healing of hydrogels

**Figure 3.**
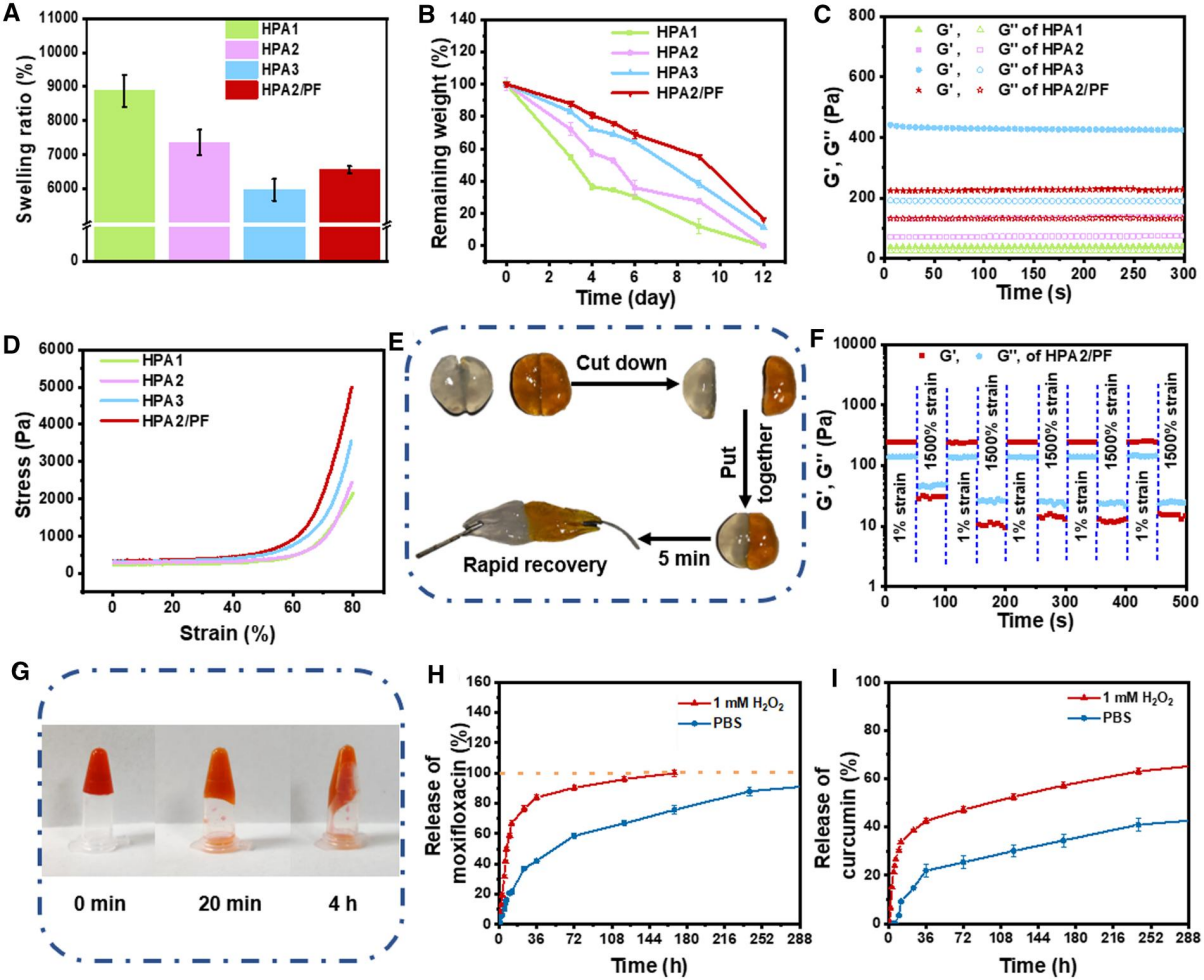
(**A**) Swelling behavior of HPA hydrogels. (**B**) Degradation behavior of HPA hydrogels. (**C**) Rheological behavior of HPA hydrogels. (**D**) The strain–stress curves of HPA hydrogels during the compression test. (**E**) Self-healing display of HPA hydrogels. (**F**) The rheological properties of HPA2/PF hydrogel when alternate step strain switched from 1% to 1500%. (**G**) ROS-responsive properties of hydrogels. The release of (**H**) moxifloxacin and (**I**) curcumin from HPA2/M&Cur-PF hydrogels in PBS or H_2_O_2_.

With the introduction of the wet healing theory [57], maintaining a certain level of humidity at the wound site is beneficial for the repair of skin wounds [58]. Figure 3A showed the equilibrium swelling ratio of HPA hydrogels, all hydrogels can absorb more than 60 times water of their own mass. The swelling ratio of HPA hydrogels decreased with increasing of PVA concentration, and their swelling ratios were 8878.8 ± 470.1%, 7360.0 ± 372.5% and 5971.1 ± 322.1% for HPA1, HPA2 and HPA3, respectively. This should be due to the increase of PVA content, which provides more crosslinkable sites and increases the crosslinking density within a certain range. Besides, the swelling ratio of HPA2/PF was 6560.5 ± 107.3%, slightly lower than that of HPA2 hydrogel, because of hydrogen bonding between PF micelles and HPA hydrogel network. The good swelling properties of the HPA hydrogels make it natural and great advantages in the management of wound exudate, and it can absorb the exudate well while maintaining the wound moist.

Biodegradability is a crucial criterion for measuring medical materials [59]. Therefore, the degradation performance of HPA hydrogels was further evaluated. Figure 3B showed that all hydrogels have good degradability. With increasing of PVA concentration, the crosslinked network of hydrogels was closer, so the degradation rate of HPA3 hydrogels was the slowest. Specifically, after 3 days of testing, the remaining weight of HPA1, HPA2 and HPA3 hydrogels groups remained 54.6 ± 1.8%, 72.0 ± 4.0%, and 83.0 ± 1.7%, respectively. Since the addition of PF micelles maked the crosslinked network of hydrogel more closely, HPA2/PF degraded more slowly than HPA2, showed 88.0 ± 1.7% remaining weight after 3 days of testing. Importantly, the remaining weight of all hydrogels was less than 30% after the 12 days test. Experiments verified that all hydrogel dressings prepared in this study had reasonable degradation properties.

Appropriate modulus is very important for hydrogel dressings. It can provide a good mechanical matching between hydrogel dressings and skin tissues, ensure a comfortable sense of wear during use, and reduce physical damage to damaged tissues. Therefore, rheological tests were used to evaluate the properties of HPA hydrogels and Figure 3C showed that with increasing of PVA concentration, the modulus of the hydrogel showed a trend of gradual increase. The storage modulus of HPA1 was 40.6 Pa, that of HPA2 was 139.1 Pa, and that of HPA3 was 424.7 Pa. Due to the addition of PF micelles, the modulus of HPA2/PF hydrogel was also increased to 227.4 Pa compared with HPA2 hydrogel. The thermal stability of the hydrogels in the range of 25–40°C was tested. As shown in the Supplementary Figure S2, the modulus of HPA2 and HPA2/PF hydrogels did not change much. In addition, we can clearly observe that the HPA2/PF hydrogel can still maintain the gel-forming state under the high temperature condition of 40°C. The hydrogel has good thermal stability between 25°C and 40°C, so when applied to the skin surface, the hydrogel can still maintain its own stability even under body temperature conditions.

Skin’s inevitable stretching during human activities is very easy to damage the hydrogel dressings. Therefore, higher requirements are put forward to the mechanical properties of hydrogels. As shown in Figure 3D, it can be seen from the stress–strain test that all hydrogels showed good compressibility. When the strain was 80%, with the increase of PVA concentration, the stress of the hydrogels was 2161.7, 2445.9 and 3547.0 Pa, respectively, and all hydrogels were unbroken. After the addition of PF micelles, the stress of HPA2/PF hydrogel increased to 4998.7 Pa at the strain of 80%. The above experimental results showed that the mechanical properties of the hydrogel gradually increase with increasing of PVA concentration, and it can be effectively improved by adding PF micelles. The results of the above tests showed that the properties of HPA hydrogels, including pore size, swelling, degradation, modulus, and mechanical properties, can be easily regulated by adjusting the ratio between components. This tunable property provides a broader selectivity for the hydrogel to adapt to different wound states and repair stages.

The damage of hydrogel dressing will lose the protective effect to wound, which puts forward requirements for the self-healing performance of skin wound dressing. As shown in Figure 3E, after the hydrogel was cut into two halves, it can quickly self-heal and merge within 5 min. Figure 3F showed that when the strain was higher than 1500%, G″ of hydrogel was higher than G′, which means that the hydrogel network was damaged. Therefore, by constantly switching low strain (γ = 1%) and high strain (γ = 1500%), the quantitative self-healing function was tested. At the beginning of the test, at the first low strain (γ = 1%), G′ and G″ was 241.3 and 138.4 Pa, respectively, and G′ > G″. After switching to the high strain (γ = 1500%), G′ changed to 27.6 Pa and G″ changed to 44.7 Pa, G″ > G′, which means that the hydrogel network crashed. In the following tests, when γ was cyclically transformed, G′ and G″ can recover to the initial value, with no significant difference in modulus compared to the first test. In conclusion, due to the existence of phenylboronic acid ester dynamic bond, the HPA hydrogel prepared in this study has good self-healing performance, which provides the quickly restore of structural integrity when it is ruptured under external force, not only ensures the physical barrier effect, but also avoids the possible bacterial invasion after rupture. As shown in the Supplementary Figure S3, shear rate scanning measurements indicate that all hydrogels in this experiment exhibit shear-thinning behavior, where the viscosity of the material depends on the shear force, and thus the hydrogels in this work are injectable.

### ROS-responsive drug release of hydrogels

The biggest problem of using antibiotics is bacterial resistance [60]. Loading drugs in hydrogels can greatly avoid the repeated use of drugs and reduce the generation of bacterial resistance. In particular, the ROS response caused by the presence of phenylboronic acid dynamic ester bond in HPA hydrogel can realize intelligent on-demand drug delivery in the hydrogel system [61]. As shown in Figure 3G, when 200 μl of 10 mM H_2_O_2_ was added to the prepared 500 μl HPA2/M&Cur-PF hydrogel, a certain fluidity of the hydrogel can be observed just within 20 min, and the hydrogel network collapsed completely at 4 h. This is mainly due to the destruction of the hydrogel network structure caused by the reaction of the phenylboronic acid ester structure with H_2_O_2_. As shown in Supplementary Figure S4, the modulus of HPA2 hydrogels and HPA2/PF hydrogels were tested under 1 mM H_2_O_2_ or PBS for the same time, respectively. At 10 min, the modulus of the hydrogel in PBS did not change much compared to the initial value, but the modulus of the hydrogel in H_2_O_2_ decreased dramatically by about 200 Pa. At 20 min, the modulus of the hydrogel in PBS decreased due to the swelling and absorbing of water, and at this time, the hydrogel in H_2_O_2_ was close to the state of non-gelation. In this study, two drugs were loaded into the hydrogel dressings. One was moxifloxacin with antibacterial effect directly mixed in the hydrogel, and the other was Cur with anti-inflammatory and antioxidant functions encapsulated in PF micelles. As shown in Figure 3H, the release rate of moxifloxacin in PBS is relatively slow, and the release time can be more than 250 h. While in 1 mM H_2_O_2_ release solution, the release amount of moxifloxacin in 36 h can reach more than 80%. The above results showed that hydrogel can deliver antibiotics quickly and effectively after being applied to wounds due to the ROS responsiveness of phenylboronic acid ester bonds. These results mean that when bacterial infection is serious, leading to excessive inflammation and the production of a large amount of ROS, the ROS response can be quickly activated, so that the antibiotic can be released rapidly to prevent the continuation of severe infection in a short time. The release amount of Cur in PBS and H_2_0_2_ was 14% and 38% at 24 h. In summary, moxifloxacin can be rapidly released more than 80% within 36 h, and Cur can be continuously released within 288 h under the action of 1 mM H_2_O_2_. In a word, the spatiotemporally sequential release of these two drugs allows for a rapid antimicrobial treatment and then sustained anti-inflammatory and antioxidant effect at the site of the infected wound.

### Antibacterial properties of hydrogels

**Figure 4.**
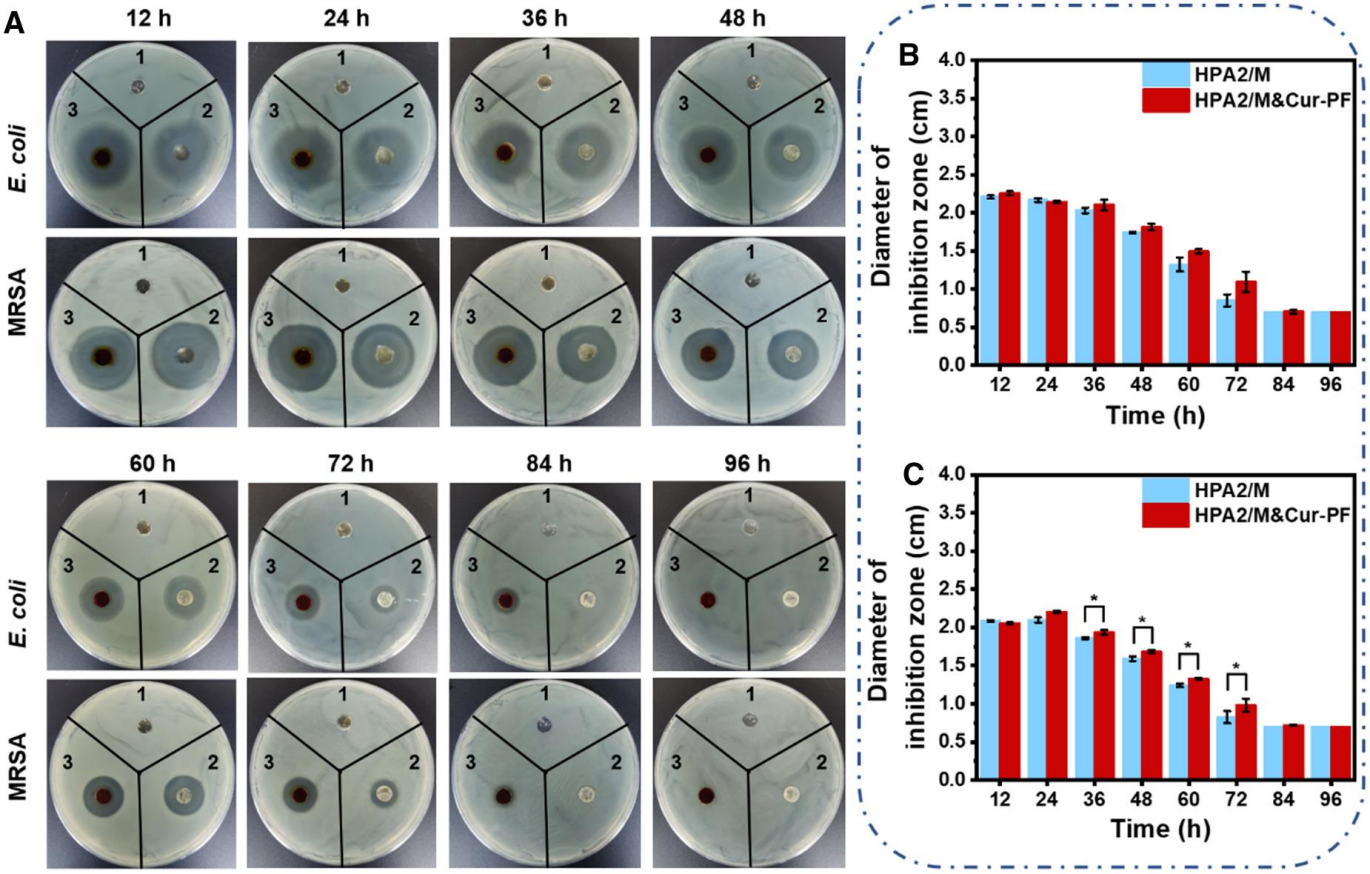
(**A**) The inhibition zone of hydrogel for *E. coli* and MRSA within 96 h. In the figure, 1, 2 and 3 represent the control group, the HPA2/M group and the HPA2/M&Cur-PF group, respectively. Statistics of inhibition zone of hydrogel for (**B**) *E. coli* and (**C**) MRSA within 96 h (*P < 0.05).

The antibacterial effect of the antibiotics released from the hydrogel was verified by the inhibition zone test. The control group is just the holes punched on the agar plate by the punch, so the diameter size and shape of the control group do not change with time and environmental factors, and at the same time to ensure that the initial diameter is the same between every group, and to confirm whether the experimental group formed a ring of inhibition around the holes. As shown in Figure 4A, at the 12 h, the diameter of the inhibition zone for HPA2/M hydrogel and HPA2/M&Cur-PF hydrogel against *E. coli* were 2.20 ± 0.02 and 2.26 ± 0.02 cm, respectively, and the diameter of the inhibition zone against MRSA were 2.08 ± 0.01 and 2.05 ± 0.01 cm, respectively. This suggested that the antibacterial effect of HPA2/M&Cur-PF hydrogels is better than that of HPA2/M hydrogels. Until the 60 h, there was still obvious inhibition zone. At this time, the diameter of HPA2/M hydrogel and HPA2/M&Cur-PF hydrogel against *E. coli* was 1.30 ± 0.09 and 1.50 ± 0.02 cm, respectively, and the diameter of the inhibition zone against MRSA was 1.24 ± 0.02 and 1.33 ± 0.01 cm, respectively. The appearance of inhibition zone indicates that moxifloxacin and Cur-PF loaded in the hydrogel diffuse to the surrounding environment, thus killing the bacteria in a certain area. In addition, the inhibition zone diameter of HPA2/M&Cur-PF hydrogel for MRSA was statistically larger than HPA2/M hydrogel, and showed significant different (*P* < 0.05). This is because Cur also has antibacterial effect [62], which is synergistic with moxifloxacin. More obviously, at the 84 h, the inhibition zone of HPA2/M hydrogel had disappeared, while the inhibition zone of HPA2/M&Cur-PF hydrogel was still slightly larger than 7 mm, suggesting that HPA2/M&Cur-PF hydrogels had better antibacterial effect than HPA2/M hydrogels. In conclusion, the hydrogel dressings prepared in this study had good sustained antibacterial effect.

### Biocompatibility of hydrogel

**Figure 5.**
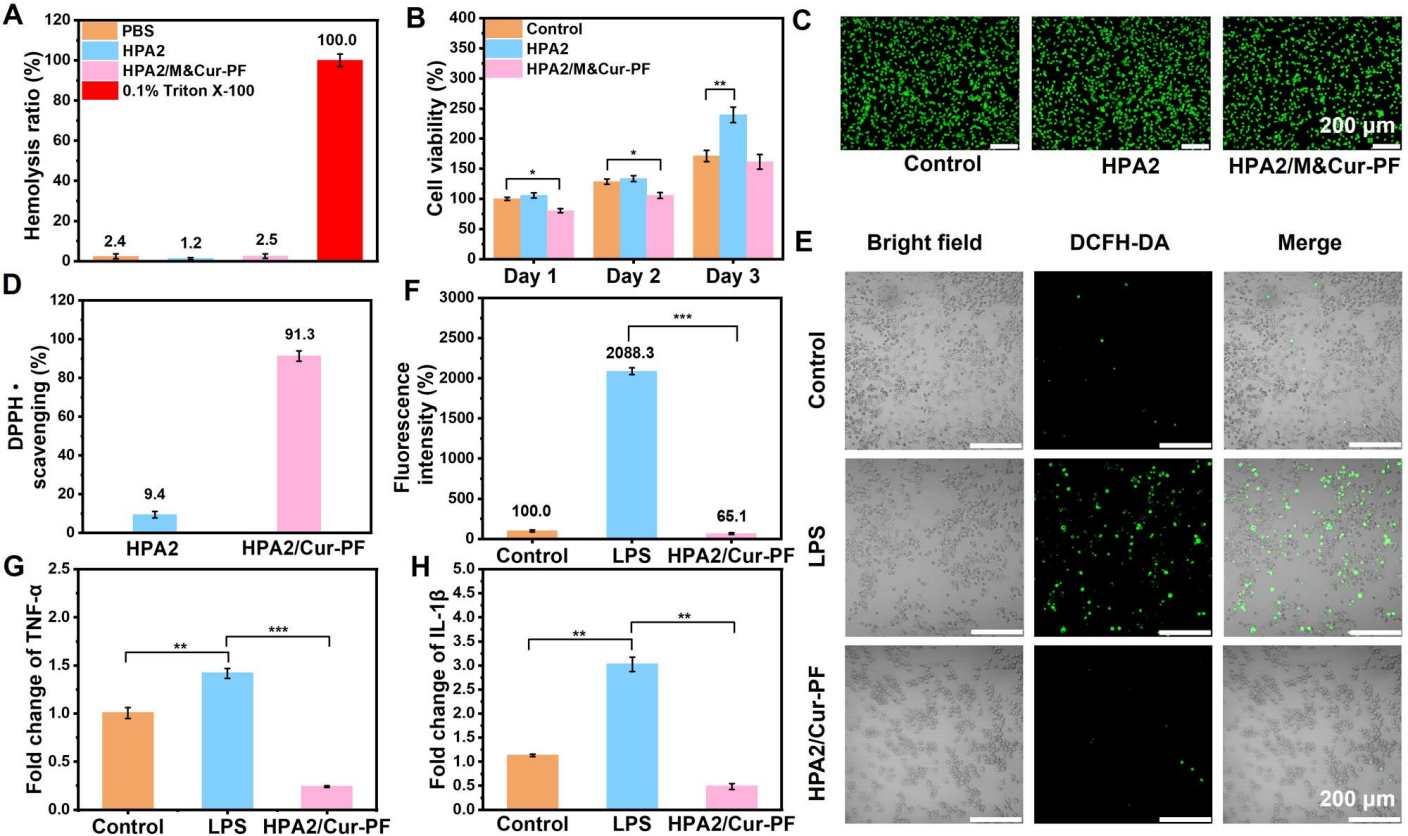
(**A**) Hemolysis ratio of hydrogel. (**B**) Cell compatibility of hydrogel on L929 cells within 3 days test. (**C**) Live/dead straining of L929 cells on the first day. (**D**) DPPH scavenging statistics of hydrogel. (**E**) Representative images of ROS scavenging experiments and (**F**) statistical results for fluorescent areas. Statistic of expression results of (**G**) TNF-α and (**H**) IL-1β after application of hydrogels on macrophages (*P < 0.05, **P < 0.01, ***P < 0.001).

Good biocompatibility is an essential prerequisite for biomedical materials [63]. In this study, the prepared hydrogels were tested from the aspects of blood compatibility and cell compatibility. As shown in Figure 5A, in the blood compatibility test, the materials in experimental group showed comparable to or even lower hemolysis ratio than that of the PBS group, with hemolysis ratio of below 5%, which was considered to be a good range of blood compatibility. The test results indicated that they will not cause significant hemolysis when applied to biological tissues. Figure 5B showed the cell compatibility of the hydrogels. Because of the good adhesion and proliferation effect of HA on cells [64], the cell viability of HPA2 hydrogel group was higher than that of the control group after co-culture. The cell viability of the HPA2/M&Cur-PF group compared to the control group was 80.3%, 82.1%, and 94.4% in the first 3 days, respectively. Although the cell viability of the experimental group was slightly decreased in the first 2 days of testing compared to the control group, the significant differences were all *P* < 0.05, indicating that the material was not toxic. Figure 5C exhibited the Live/Dead staining images of L929 cells after co-cultured with the hydrogel leachate for one day, which is consistent with the quantitative statistical results of cell viability. In general, the hydrogel dressings prepared in this study had good biocompatibility.

### Antioxidation and anti-inflammation of hydrogel

If MRSA-infected wounds are not treated in a timely and appropriate manner, it can easily change to severe infection, causing severe inflammation at the wound site, and producing a large amount of ROS through oxidative stress [65, 66]. Therefore, we first used the scavenging experiment of stable free radical DPPH• to evaluate the antioxidant properties of the hydrogels. Figure 5D showed that the presence of PBA group brings little DPPH• scavenging capacity to the hydrogels. Furthermore, due to the good antioxidant capacity of Cur, 500 μl of HPA2/Cur-PF hydrogel can scavenge more than 90% of DPPH. In addition, Figure 5E further showed the ROS scavenging ability of the hydrogels by DCFH-DA staining. LPS-induced macrophage (RAW 264.7) produced a large amount of ROS, while normal macrophage (RAW 264.7) cells in the control group only produced a small amount of ROS and the HPA2/Cur-PF hydrogel group showed similar or even lower ROS intensity than the control group. The statistical results in Figure 5F showed that the ROS production in the experimental group was not significantly different from that in the control group. As is well known, high concentrations of ROS can cause DNA damage and cell death, while low concentrations of ROS play a crucial role in signal transduction and wound repair [67]. Therefore, the experimental group didn’t show a significant different amount of ROS compared to the control group, which is very beneficial for wound repair. As shown in Figure 5G and H, the significantly increased expression levels of TNF-α and IL-1β in the LPS group indicate that macrophage (RAW 264.7) cells were successfully induced into an inflammatory state, while the expression levels of both inflammatory factors were significantly reduced under the action of the hydrogel group, indicating that the hydrogel has significant anti-inflammatory effects. In summary, the HPA2/Cur-PF hydrogels have suitable antioxidant and anti-inflammatory effects, which lays a strong foundation for its use as a dressing for wound with MRSA infection.

### Promoting effect of HPA/M&Cur-PF hydrogel on healing of MRSA-infected skin wounds

**Figure 6.**
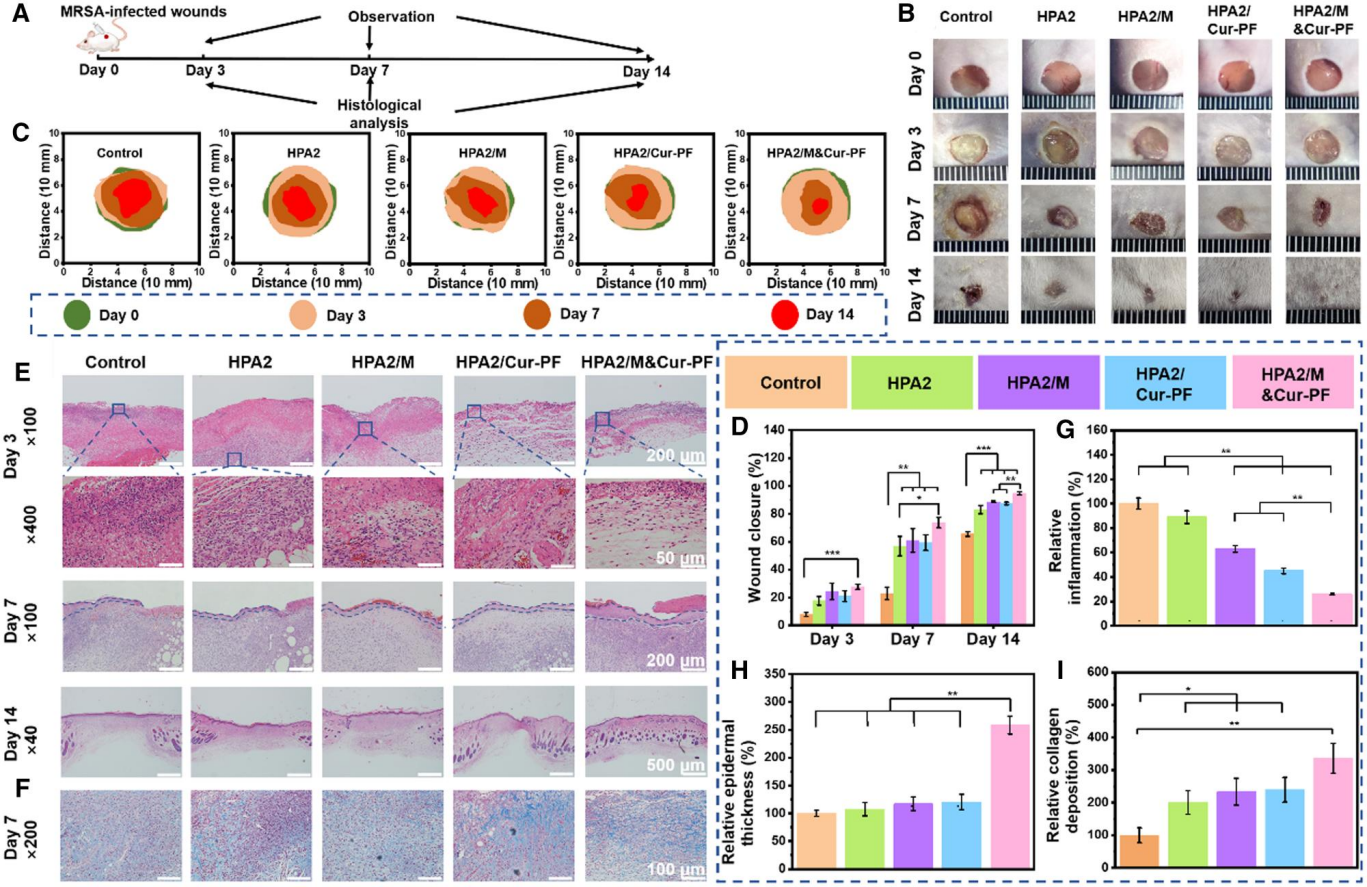
(**A**) Schematic diagram of the *in vivo* wound healing experimental program in an infected full-thickness skin defect model. (**B**) The pictures of wounds on the 3rd, 7th, and 14th day were divided into five groups: Tegaderm™ film dressing (control), HPA2 hydrogel, HPA2/M hydrogel, HPA2/Cur-PF hydrogel and HPA2/M&Cur-PF hydrogel, and (**C**) plotting of wound area over time. (**D**) Statistics on changes in wound closure ratio. (**E**) HE staining of the wound site on the 3rd, 7th, and 14th day. (**F**) Masson staining of the wound site on the seventh day. (**G**) Statistics of relative inflammation at the wound site on the third day. (**H**) Thickness of regenerated epidermis at the wound site on the seventh day. (**I**) Statistics of relative collagen deposition at the wound site on the seventh day (*P < 0.05, **P < 0.01, ***P < 0.001).

A series of *in vitro* experiments have verified the good antibacterial, anti-inflammatory and antioxidant effects of HPA/M&Cur-PF hydrogel. We further established a MRSA-infected mouse back skin wound model, and evaluated the repair-promoting effect of the hydrogel prepared in this study. Figure 6A showed the entire time course from modeling to wound repair. First, a circular wound with a diameter of 8 mm was created on the back skin of mouse, and 10 μl of 10^8^ CFU/ml MRSA were injected. On the 3rd, 7th, and 14th day, the skin at the wound site was observed and sectioned for study. The commercially available Tegaderm™ dressing was selected as the control group [68]. Based on the previous characterization tests, HPA2 hydrogel was selected as the representative and applied in the subsequent animal experiments. The experimental groups were HPA2 hydrogel, HPA2/M hydrogel loaded with moxifloxacin (the concentration of moxifloxacin was 2 mg/ml), HPA2/Cur-PF hydrogel loaded with Cur-encapsulated PF (the concentration of Cur was 2 mg/ml), and HPA2/M&Cur-PF hydrogel loaded with moxifloxacin and Cur-encapsulated PF (the concentration of moxifloxacin and Cur were both 2 mg/ml). In Figure 6B, the wounds in each group showed different phenomena over time after surgery. Control group showed significant suppuration on the third day after treatment, which even lasted until the seventh day. This is consistent with the clinical phenomenon that MRSA-infected wounds have severe bacterial infection with a lot of inflammation and even difficulty in healing. The HPA2 hydrogel group did not have a good anti-infection effect compared to the other three experimental groups, and no significant sepsis was found. In Figure 6C, the wound areas of each group were plotted over time. In Figure 6D, the wound closure ratio of each group was analyzed. Compared with the control group, after 7 days of treatment, there was a significant difference (*P* < 0.01) in the wound closure of HPA2, HPA2/M, HPA2/Cur-PF, and HPA2/M&Cur-PF group, respectively. Besides, there was a significant difference in the wound closure of HPA2/M&Cur-PF and HPA2 (*P* < 0.05), which indicated that the hydrogel loaded with the two drugs promoted better wound repair. On the 14th day, there still was a significant difference (*P* < 0.01) between HPA2/M&Cur-PF and HPA2/M and HPA2/Cur-PF, which proved that moxifloxacin and Cur promoted MRSA-infected wound healing, and the synergistic effect of the two drugs was more favorable for wound repair. In conclusion, on the 14th day, all hydrogel groups showed good healing effect. The HPA2/M&Cur-PF hydrogel group showed the best healing effect, with almost complete wound closure. But the control group still had an obvious wound, with 34.3% of the remaining wound area, which was significantly different from the other hydrogel groups (*P* < 0.001).

The effect of each group on wound repair was further evaluated by HE staining. In Figure 6E, on the third day, a large amount of inflammation can be seen in the control group, while the inflammation in other hydrogel groups is relatively low. Figure 6G showed the statistics of inflammation. Due to the effects of moxifloxacin and Cur, the inflammation of HPA2/M, HPA2/Cur-PF and HPA2/M&Cur-PF hydrogel groups is significantly lower than that of the control group and HPA2 hydrogel group (*P* < 0.01). The dual effect of antibacterial and anti-inflammatory effects makes the relative amount of inflammation between HPA2/M&Cur-PF hydrogel group and HPA2/M and HPA2/Cur-PF have a significant difference (*P* < 0.01). In Figure 6E, on the seventh day, the epidermal regeneration in the control group was discontinuous, accompanied by blood scabs, which was mainly because the wound site was infected by MRSA and the bacteria were not removed in time, and the infection also made it difficult for wound healing to continue the next step. However, obvious continuous epidermal regeneration was seen in HPA2/M, HPA2/Cur-PF and HPA2/M&Cur-PF hydrogel groups. In Figure 6H, statistics showed that the thickness of regenerated epidermis in HPA2/M&Cur-PF hydrogel group was the thickest, which was significantly different from the other four groups (*P* < 0.01). The metabolism of collagen participates in the whole process of wound repair and the regenerated collagen constitutes an important part of the repaired wound, so its importance is obvious. In Figure 6F, masson staining showed the deposition of collagen in each group on the seventh day. And in Figure 6I, a statistical analysis of the masson staining was performed. The relative collagen deposition of the four test groups was significantly higher than that of the control group, and the difference was significant compared with the control group (*P* < 0.05). Among the experimental groups, the HPA2/M&Cur-PF group, which was more effective for wound repair, had 3.4 times more collagen deposition than the control group. In summary, HPA2/M&Cur-PF hydrogel dressings can promote wound closure, reduce inflammation and promote collagen deposition in MRSA-infected mouse skin wound healing.

### Immunofluorescence staining analysis

**Figure 7.**
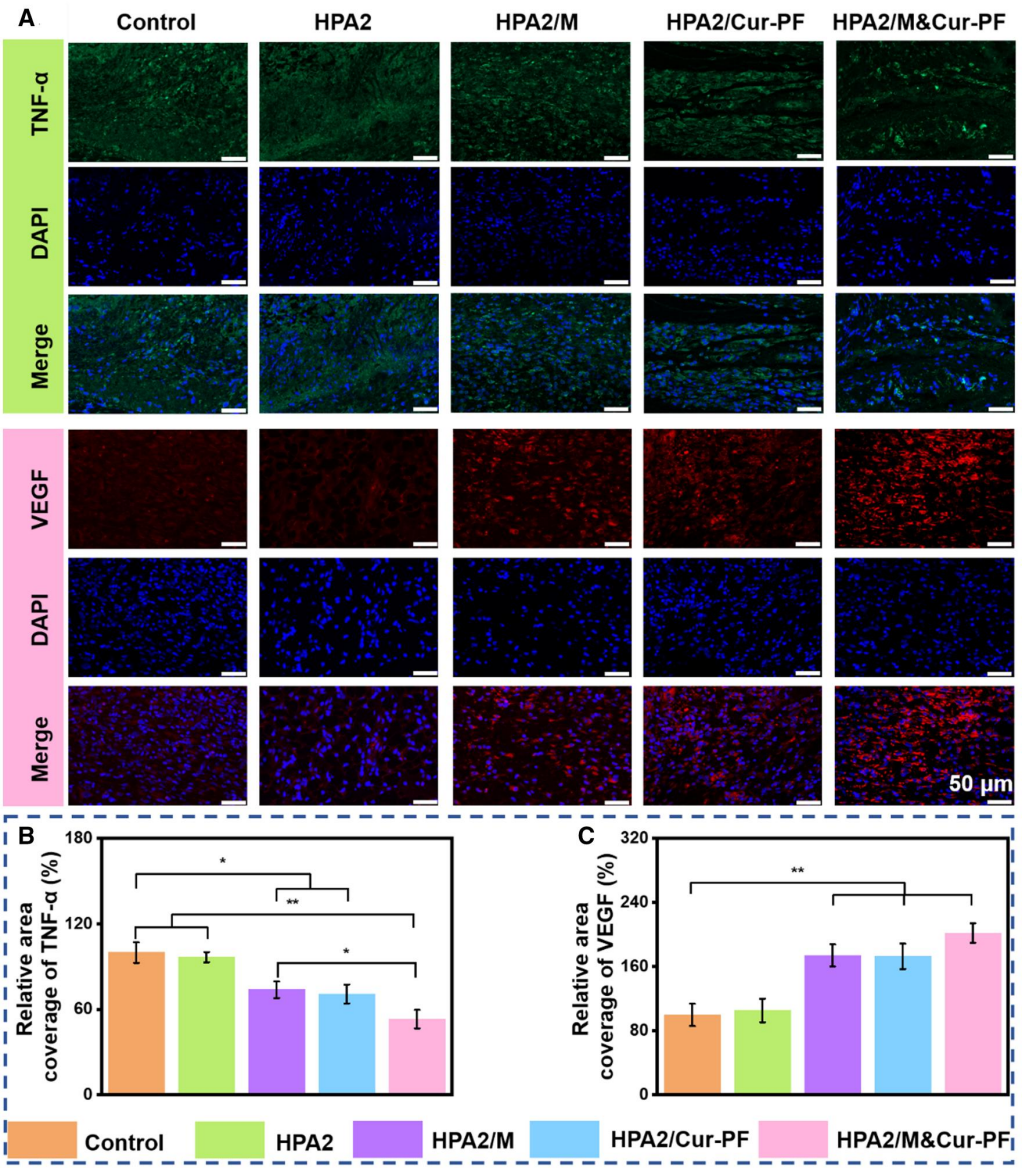
(**A**) Immunofluorescence staining images of TNF-α on the third day and VEGF on the seventh day, with green indicating TNF-α expression and red indicating VEGF. (**B**) Quantitative analysis of the relative area percentage (*n* = 3) of TNF-α and (**C**) VEGF. For all quantitative analyses, the commercial film group data for TNF-α on the third day and VEGF on the seventh day were set as 100% (*P < 0.05, **P < 0.01).

Wound healing is a complex process, and the expression of relevant cytokines can reflect some specific situations during the wound healing process [69, 70]. The third day after wound formation is considered to be the inflammatory period. At this time, a proinflammatory cytokine, tumor necrosis factor (TNF-α) [71], was selected to evaluate the effect of hydrogel on inflammation control. From the immunofluorescence staining analysis of TNF-α in Figure 7A and the statistics in Figure 7B, it can be seen that the colonization of bacteria at the wound leads to the aggravation of inflammation, and there were significant differences between control and HPA2/M, HPA2/Cur-PF and HPA2/M&Cur-PF hydrogel groups, respectively. Specifically, inflammation in HPA2/M and HPA2/Cur-PF hydrogel groups was significantly reduced. The reduction in inflammation in the HPA2/M hydrogel was due to the antibacterial effect of moxifloxacin, while the reduction in inflammation in HPA2/Cur-PF hydrogel was due to the antibacterial and anti-inflammatory effects of Cur. Most importantly, HPA2/M&Cur-PF hydrogel group has the synergistic effect of two drugs, so it showed the lowest inflammation. The generation of blood vessels is an essential physiological process in wound healing, which can rebuild normal blood flow for wound tissue, provide nutrition and oxygen for the tissue, and accelerate the process of wound repair [72, 73]. On the seventh day, vascular endothelial growth factor (VEGF) [74] was used to evaluate the neovascularization at the wound. Figure 7A showed the immunofluorescence staining analysis of VEGF and Figure 7C showed the statistical results. Significant differences (*P* < 0.01) were found between the control group and the HPA2/M hydrogel group, the HPA2/Cur-PF hydrogel group and the HPA2/M&Cur-PF hydrogel group, respectively. In HPA2/M and HPA2/Cur-PF hydrogel groups, due to the effect of two drugs to avoid bacterial infection and inflammation in the early stage, the hydrogel promoted the formation of new blood vessels. In conclusion, based on the expression of TNF-α and VEGF, HPA2/M&Cur-PF hydrogel showed better therapeutic effect in MRSA-infected skin wounds.

## Conclusion

In this study, HA-based HPA/M&Cur-PF hydrogel dressing with spatiotemporally sequential delivery of antibacterial and anti-inflammatory drugs was constructed based on the dynamic bond of phenylboronic acid ester for the first time, and was used to repair MRSA-infected skin wounds. The rheological properties, self-healing, biocompatibility, responsiveness, spatiotemporally sequential drug delivery, antibacterial, antioxidant and anti-inflammatory properties of hydrogels were verified. Under ROS conditions, hydrogels can release more than 80% of moxifloxacin within 36 h to perform quickly anti-infection effect, and release Cur up to 288 h to perform a sustained anti-inflammation effect. Finally, in the MRSA-infected mouse skin wound healing, the hydrogel-treated group exhibited faster wound closure, reduced inflammation, and promoted epidermal growth and collagen deposition. Immunofluorescence staining results also demonstrated the hydrogels’ ability to reduce inflammation while also promoting angiogenesis. In conclusion, HPA/M&Cur-PF hydrogel dressing has a significant effect on the repair of skin wounds infected by MRSA, providing ideas for responsive spatiotemporally sequential drug delivery strategies.

## Supplementary Material

rbad110_Supplementary_Data
