# Supplementary material for: ROS-responsive hydrogels with spatiotemporally sequential delivery of antibacterial and anti-inflammatory drugs for the repair of MRSA-infected wounds
Source: Regen Biomater. 2023 Dec 9;11:rbad110. doi: 10.1093/rb/rbad110 (PMC10761208; doi:10.1093/rb/rbad110)
Supplement: rbad110_Supplementary_Data [file rbad110_supplementary_data.zip › Supporting Information.pdf]

Supporting Information

ROS-responsive hydrogels with time-space sequential delivery of antibacterial and anti-inflammatory drugs for the repair of MRSA-infected wounds

Characterizations

^{1}H NMR spectra of hyaluronic acid grafted 3-aminophenylboronic acid (HA-PBA) was performed using a Bruker Ascend 400 MHz NMR instrument with deuteroxide serving as the solvent.

FT-IR spectra of hyaluronic acid (HA), 3-aminophenylboronic acid (PBA), and hyaluronic acid grafted 3-aminophenylboronic acid (HA-PBA) were recorded in the range of 2000-650 cm^{-1} by employing a Nicolet 6700 FT-IR spectrometer (Thermo Scientific Instrument).

The morphologies of PF127 micelle were examined by a transmission electron microscope (FEI, Tecnai, F20FEG-TEM). One drop of polymer solution in DI water was cast on a carbon-coated copper grid and the water was then evaporated for 6 h in a vacuum oven before test [1].

Sample preparation for SEM may cause artifacts and can have effects on pore size. However, freeze drying of hydrogel is most widely used sample preparation method for SEM observation [2]. The morphologies of freeze-dried hydrogels were examined by a field emission scanning electron microscope (FE-SEM; QUTAN FEG 250, FEI) after sprayed with a thin gold layer. NIH Image J software was used to measure the pore diameters of hydrogel samples. For each hydrogel sample (at least 3 samples), every

hydrogel had four pictures taken from various regions of the hydrogel. Every picture was measured by more than five pores.

Swelling and degradation test

Swelling test was performed to determine the equilibrium swelling ratio (ESR) and stability of the hydrogels. The completely gelled wet hydrogels were put into 20 mL PBS (0.01 M pH 7.4) in sealed vials at 37 °C with shaking at 100 rpm. The test was not finished until the weight of all hydrogels kept constant. Following that, the hydrogels were weighed. ESR was calculated using the following equation: ESR= $(W_t-W_i)/W_i$, where W_i and W_t represented the initial dry weight and after swelling weight, respectively [3]. After the swelling reaches equilibrium, the test was not finished until all the hydrogels were degraded completely. The degradation rate was calculated using the following equation: Weight remaining (%) = $(W_{t2} - W_{i2})/W_{i2}$, where W_{i2} and W_{t2} represented the initial weight of the wet hydrogels and the wet weight at the pre-set time point, respectively. Each group contains three replicate samples.

Rheological property of the hydrogels

The rheological test of these hydrogels was carried out by using a TA rheometer (DHR-2). Time sweep test with 1% constant strain and a constant frequency of 10 rad/s at 37 ℃ was used to evaluate the stiffness of these hydrogels. Hydrogel discs with a diameter of 20 mm and a thickness of 1000 μm were used as samples for testing, and the periphery was sealed by silicone oil to prevent the evaporation of water [4].

Mechanical performance

The mechanical performance of the hydrogels was tested by compression test using TA

rheometer (DHR-2) at room temperature. The hydrogels were fabricated as cylindrical shapes with a diameter of 8 mm and height of 10 mm. The compression test of the hydrogels were conducted with the maximal compression strain up to 80% at a strain speed of 100 μm/s [3].

Self-healing performance of hydrogels

The hydrogel disks were prepared with a 20 mm diameter and 1 mm height. Using the strain amplitude sweep method (γ from 1% to 1500%), the value of the critical strain region was recorded. Then the other hydrogel disks were employed to test the self-healing behaviors by alternate strain sweep test at a fixed angular frequency (1 $rad \cdot s^{-1}$). Amplitude oscillatory strains were switched from small strain (γ = 1.0%, 50 s for each interval) to large strain (γ = 1500%, 50 s for each interval), and 5 cycles were carried out.

In vitro drug release assay

The drug release characteristics of HPA/M&Cur-PF hydrogels were tested in PBS and 1 mM H_2O_2 for moxifloxacin and curcumin. First, 200 μL of HPA/M&Cur-PF hydrogel was prepared in a 4 mL centrifuge tube. After complete gelation, the drug was encapsulated in the hydrogel in situ. Subsequently, 3 mL of PBS or 1 mM of H_2O_2 were added to the centrifuge tubes, and the tubes were placed at 37 °C. The centrifuge tubes were then incubated for a specified period of time, and placed in a shaker at 37 °C. After the specified time, 1 mL of release solution is removed for further analysis. Then 1 mL of fresh buffer was added to the tube to maintain a constant volume. Finally, the drug released from the hydrogel was analysed by UV-Vis spectrophotometer at 420 nm

(Curcumin) and 288.57 nm (Moxifloxacin), respectively. [5]

Antibacterial property test of the hydrogels

In this assay, agar plates were spread with 150 μL of bacterial suspension (10^8 CFU/mL). The samples were placed on solid medium (nutrient agar) in contact with the bacteria and the zones of inhibition around each sample were measured to record the antibacterial effect of HPA/M hydrogel loaded with moxifloxacin, and HPA/M&Cur-PF hydrogel loaded with moxifloxacin and curcumin. The hydrogels were placed on agar plates and incubated at 37 °C for 12 h. After 12 h, the hydrogels were transferred to new agar plates covered with bacteria and incubated for another 12 h. Subsequently, the above procedure was repeated until no zone of inhibition appeared on the new agar plate [6].

Antioxidant efficiency of hydrogels

The antioxidant efficiency of hydrogels was evaluated by the method of scavenging the stable 1, 1-diphenyl-2-picrylhydrazyl (DPPH) free radical [7]. The hydrogels were cut into homogenate by using tissue grinder. DPPH solution was prepared under light-avoiding conditions by adding 3.94 mg of 2,2-diphenyl-1-(2,4,6-trinitrophenyl) hydrazide to 10 mL ethanol. Afterwards, 500 μL hydrogel and 300 μL DPPH solution were added to 2.7 mL ethanol. The mixture was stirred and incubated in a dark place for half an hour. Next, the wavelength of DPPH was scanned by a UV–vis spectrophotometer. The degradation of DPPH was calculated by the following formula:

$$\text{DPPH scavenging \%} = \frac{A_B - A_H}{A_B} \times 100$$

where A_B, A_H are the absorption of the blank (DPPH + ethanol) and the absorption of

the hydrogel (DPPH +ethanol +hydrogel), respectively.

In vitro ROS scavenging test of the hydrogels

2×10^4 mouse-derived macrophages (RAW264.7) were placed in 24-well cell culture plates. Macrophage polarisation was induced by adding 2 μg/mL lipopolysaccharide (LPS) to each well for 12 h. No LPS was added as a control group, and the experimental group was the LPS+HPA/Cur-PF hydrogel group (hydrogel leachate, the same concentration of LPS as that of the positive control group). After 48 hours of incubation, the cells were washed 3 times with PBS, and then the DCFH-DA probe (20 μM) was added. Fluorescence in RAW264.7 cells was observed using a confocal fluorescence microscope (OLYMPUS, IX73). ROS fluorescence intensity data were obtained from at least three images of each sample using Image J software [8].

Anti-inflammatory experiments with hydrogels

5×10^4 mouse-derived macrophages (RAW 264.7) were placed in 12-well cell culture plates. The experiments were divided into control group (normal medium), lipopolysaccharide (LPS) group (LPS concentration of 100 ng/mL, positive control), and LPS+HPA/Cur-PF hydrogel group (hydrogel leachate with the same LPS concentration as that of the positive control group). The macrophage polarisation was induced at a concentration of 1 μg/mL of LPS for 12 hours. Then, all culture solutions were replaced with 10% foetal bovine serum + DMEM for the control and LPS groups, and leachate of HPA/Cur-PF hydrogel + DMEM + 10 foetal bovine serum for the hydrogel experimental group. After 48 h of incubation, total cellular RNA from macrophages was isolated using Trizol reagent (Life technologies). Then reverse

transcription was performed by reverse transcription system (Roche). Quantitative real-time polymerase chain reaction (qRT-PCR) was performed on an Applied Biosystems 7500 rapid real-time PCR system and iTaq universal SYBR Green supermix. ΔΔCt method was used for gene expression analysis [9]. GAPDH was used as a reference gene. The primer sequences used were as follows:

Gene	Apstream primer sequence	Downstream primer sequence
GAPDH	TGTGTCCGTCGTGGATCTGA	TTGCTGTTGAAGTCGCAGGAG
TNF-α	ACTCCAGGCGGTGCCTATGT	GTGAGGGTCTGGGCCATAGAA
IL1-β	AAACGGTTTGTCTTCAACAAGATAG	ATTCCATGGTGAAGTCAATTATGTC

Blood compatibility test of the hydrogels

Blood compatibility test was conducted according to reference [10]. Erythrocytes were separated by centrifugation (at 1,000 rpm) from the mice blood for 10 minutes. The obtained erythrocytes were washed three times with PBS and then diluted to a final concentration of 5% (v/v). Hydrogel (500 μL) with erythrocytes stock (500 μL) was added to a 24-well microplate, then shaken in an incubator at 37 °C for 1 h with a shaking speed of 150 rpm. After that, the microplate well contents were centrifuged (at 1,000 rpm) for 10 minutes and the supernatant (100 μL) was then introduced into a 96-well microplate. The absorbance of the solution was read at 540 nm by a microplate reader (Molecular Devices). 0.1% Triton x-100 was used as the positive control while PBS was used as the negative control. The hemolysis percentage was calculated from the relation: Hemolysis (%) = $[(A_p - A_b)/(A_t - A_b)] \times 100\%$ where A_p was the absorbance value for the experiment group, A_t was the absorbance value for the Triton x-100 positive control and A_b was the absorbance value for PBS.

Cytocompatibility evaluation of the hydrogels

The mouse fibroblast cells (L929 cells) were employed to evaluate the cytotoxicity of all hydrogels using the leaching pattern [11]. The complete growth medium was Dulbecco's modified Eagle's medium (DMEM) (Gibco) supplemented with 10% fetal bovine serum (Gibco), 1.0×10^5 U/L penicillin (Hyclone) and 100 mg/L streptomycin (Hyclone). The sterilized samples (2.5-10 mg/mL) were incubated in DMEM cultural media with penicillin included for 24 hours to prepare the leaching solutions. L929 cells were seeded in a 96-well plate with a density of 20000 cells per well and cultured in a humidified atmosphere containing 5% CO_2 at 37°C. After being cultured for 24 h, the culture medium was removed and replenished with the leaching solutions of the hydrogels (with FBS added). The cell proliferation and viability were evaluated by AlamarBlue® assay and LIVE/DEAD® Viability/Cytotoxicity Kit assay, respectively. After being co-incubated for 24 h, the leaching solution was removed and 10 μL of AlamarBlue® reagent in 100 μL complete growth medium was then added into each well. The plate was incubated for 4 h in a humidified incubator containing 5% CO_2 at 37°C. After that, 100 μL of the medium in each well was transferred into a 96-well black plate (Costar). Fluorescence was read using 560 nm as the excitation wavelength and 600 nm as the emission wavelength using a microplate reader (Molecular Devices) according to the manufacturer's instructions. Cells seeded on TCP with complete growth medium served as the control group. Each group contains five replicate samples. An inverted fluorescence microscope (IX53, Olympus) was employed to observe the cell morphology and viability.

In vivo MRSA-infected full-thickness wound healing evaluation of the hydrogels

To further study the wound healing effect of the hydrogels toward severely infected wounds, MRSA infected full-thickness skin defect model on the back area of the mice was established. Our animal experiments were approved by the institutional review board of Xi'an Jiaotong University. Male Kunming mice weighing 35-40 g and 5-6-week age were used for studies. All mice were acclimatized for 1 week priorto surgery and then randomly divided into five groups with each group containing 24 mice [12]. The five groups were as follows: Tegaderm^{TM} film (control), HPA2 hydrogel, HPA2/M hydrogel, HPA2/ Cur-PF hydrogel and HPA2/M&Cur-PF hydrogel.

The mice were anesthetized by intraperitoneal injection of chloral hydrate (10 wt%, 3mL/kg body weight), and they were shaved in the dorsal region between tail and back. One round-shaped full thickness wound with 8 mm in diameter were created on the back area using scissors and forceps, and 10 μL Methicillin-resistant Staphylococcus aureus solution (10^8 CFU/mL) was injected into the wound to establish infection. The wounds on each group were then dressed by corresponding wound dressings. For wound area monitoring, on the 3rd, 7th, and 14th day, the wound area was photographed and calculated by Image J.

Histology and immunohistochemistry

For evaluation of epidermal regeneration and inflammation in the wound area, samples collected on 3rd, 7th and 14th day were fixed with 4% paraformaldehyde for 1 h, then embedded in paraffin and cross-sectioned to 4 μm thickness slices, and then stained with Haematoxylin-Eosin or Masson trichrome. All slices were analyzed and photo-

captured by microscope (IX53, Olympus, Japan). The regenerated skins from the wound site were also excised at 3th, 7th, and 14th days for immunofluorescence staining. The fixed and frozen sections were stained with TNF-α and VEGF. The nuclei were stained with DAPI containing mounting solution. Slides were observed under an inverted fluorescence microscope (IX53, Olympus).

Results

Hydrogels	Average gelation time (s)
HPA2	50
HPA2/PF	70

Tbl. S1. Average gelation time of HPA2 and HPA2/PF hydrogels.

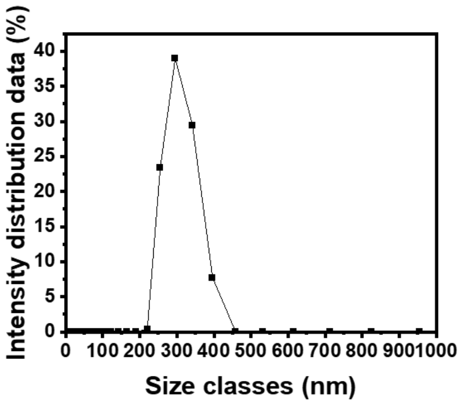

Fig. S1. The Dynamic Light Scattering (DLS) of Cur-PF.

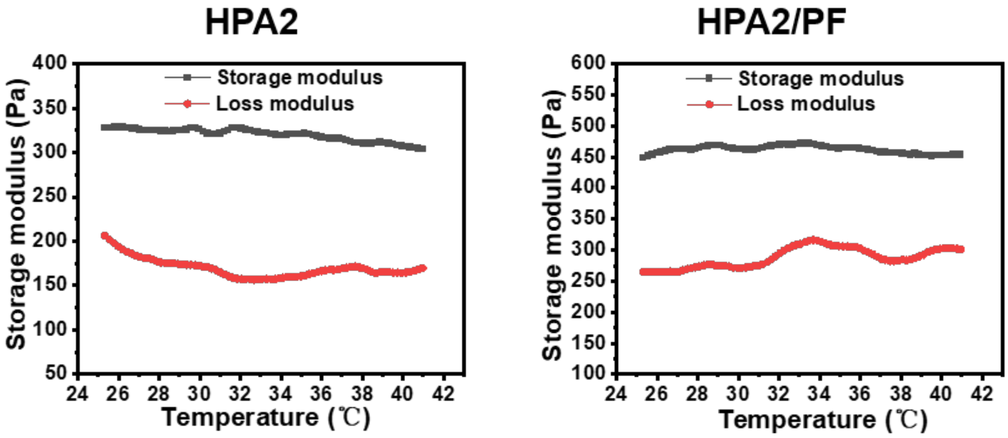

Fig. S2. The thermal stability of the HPA2 and HPA2/PF hydrogels in the range of 25°C-40°C.

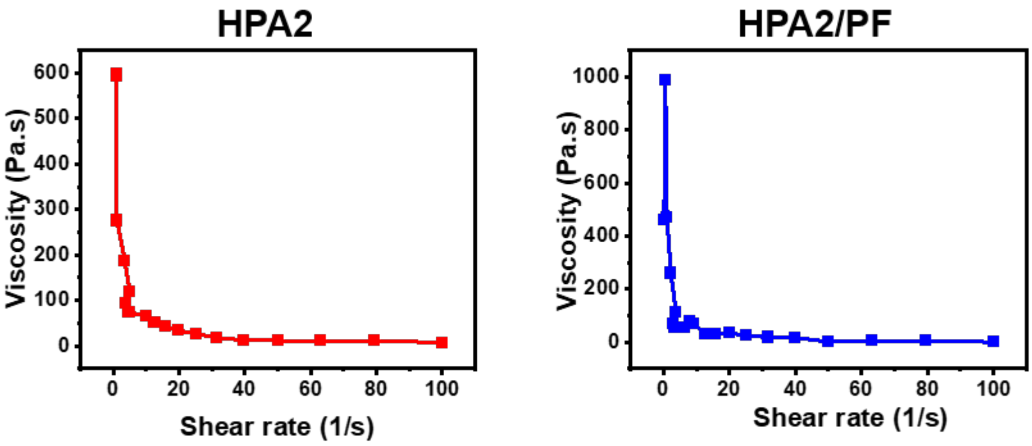

Fig. S3. The shear-thinning behavior of the HPA2 and HPA2/PF hydrogels.

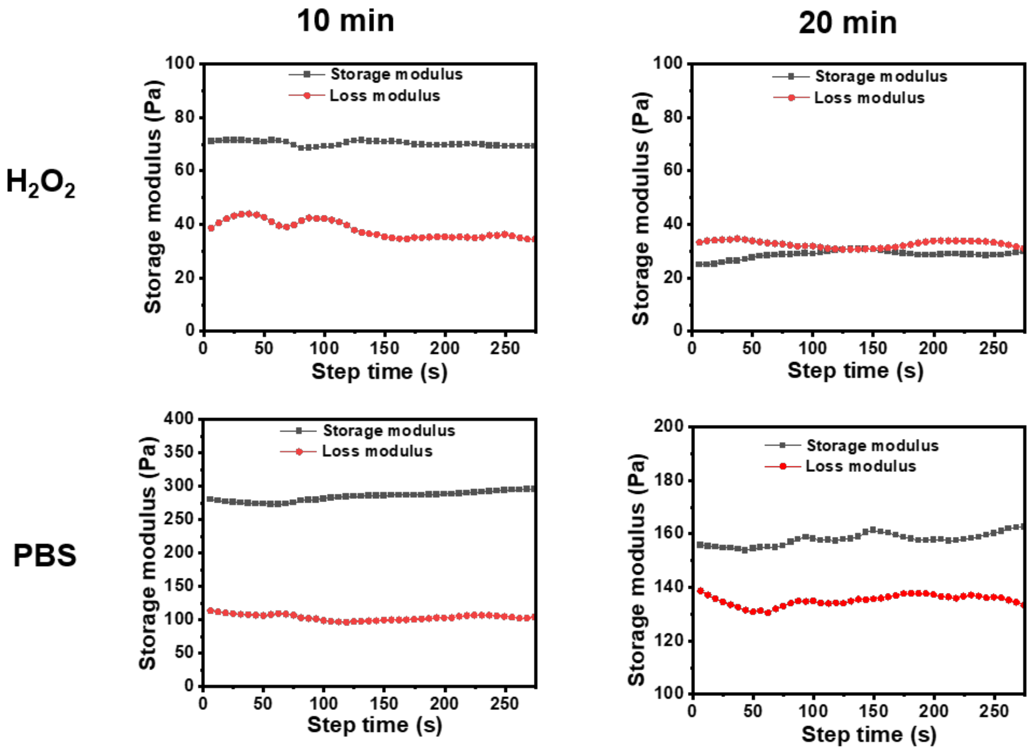

Fig. S4. Rheological behavior of HPA2 and HPA2/PF hydrogels immersed in 1 mM H_2O_2 and PBS at 10 min and 20 min.

Reference

1. Qu J, Zhao X, Liang Y, Zhang T, Ma P X, Guo B. Antibacterial adhesive injectable hydrogels with rapid self-healing, extensibility and compressibility as wound dressing

for joints skin wound healing. Biomaterials **2018**;183:185-199.

2. Liang Y, Li M, Huang Y, Guo B. An integrated strategy for rapid hemostasis during tumor resection and prevention of postoperative tumor recurrence of hepatocellular carcinoma by antibacterial shape memory cryogel. Small **2021**;17:2101356.

3. Zhao X, Dong R, Guo B, Ma P X. Dopamine-incorporated dual bioactive electroactive shape memory polyurethane elastomers with physiological shape recovery temperature, high stretchability, and enhanced C2C12 myogenic differentiation. ACS Appl Mater Interfaces **2017**;9:29595-29611.

4. Qu J, Zhao X, Ma P X, Guo B. Injectable antibacterial conductive hydrogels with dual response to an electric field and pH for localized "smart" drug release. Acta Biomater **2018**;72:55-69.

5. Liang Y, Li M, Yang Y, Qiao L, Xu H, Guo B. pH/glucose dual responsive metformin release hydrogel dressings with adhesion and self-healing via dual-dynamic bonding for athletic diabetic foot wound healing. ACS nano **2022**;16:3194-3207.

6. Liang Y, Zhao X, Hu T, Han Y, Guo B. Mussel-inspired, antibacterial, conductive, antioxidant, injectable composite hydrogel wound dressing to promote the regeneration of infected skin. J Colloid Interface Sci **2019**;556:514-528.

7. Li M, Pan G, Yang Y, Guo B. Smart aligned multi-layered conductive cryogels with hemostasis and breathability for coagulopathy epistaxis, nasal mucosal repair and bleeding monitoring. Nano Today **2023**;48:101720.

8. Wang X, Wu B, Zhang Y, Dou X, Zhao C, Feng C. Polydopamine-doped supramolecular chiral hydrogels for postoperative tumor recurrence inhibition and

simultaneously enhanced wound repair. Acta Biomater **2022**;153:204-215.

9. Sunnapu O, Khader R, Dhanka M, Kumar Vemula P, Karuppannan S. Enzyme-Responsive Hydrogel for Delivery of the Anti-Inflammatory Agent Zingerone. ChemNanoMat **2022**;8:e202200334.

10. Sasidharan A, Panchakarla LS, Sadanandan AR, Ashokan A, Chandran P, Girish C M, Menon D, Nair SV, Rao C, Koyakutty M. Hemocompatibility and macrophage response of pristine and functionalized graphene. Small **2012**;8:1251-1263.

11. Zhao X, Liang Y, Huang Y, He J, Han Y, Guo B. Physical double-network hydrogel adhesives with rapid shape adaptability, fast self-healing, antioxidant and NIR/pH stimulus-responsiveness for multidrug-resistant bacterial infection and removable wound dressing. Adv Funct Mater **2020**;30:1910748.

12. Yu R, Li Z, Pan G, Guo B. Antibacterial conductive self-healable supramolecular hydrogel dressing for infected motional wound healing. Sci China: Chem **2022**;65:2238-2251.
