# Supplementary material for: ROS-responsive hydrogels with spatiotemporally sequential delivery of antibacterial and anti-inflammatory drugs for the repair of MRSA-infected wounds
Source: Regen Biomater. 2023 Dec 9;11:rbad110. doi: 10.1093/rb/rbad110 (PMC10761208; doi:10.1093/rb/rbad110)
Supplement: rbad110_Supplementary_Data [file rbad110_supplementary_data.zip › Response to the reviewers’ comments.docx]

**Response to the reviewers’ comments**

Dear Editor and Reviewers,

We greatly thank you for these professional and constructive comments about our manuscript. These comments are valuable and helpful for revising and improving our review. Following the reviewers’ comments and suggestions, we have made extensive modifications in our manuscript to make our review more comprehensive. The modifications were highlighted in red text in the manuscript.

**List of responses to reviewers’ comments**

Reviewer: 1
Comments to the Author
Qiao et al.’s manuscript entitled "ROS-responsive hydrogels with spatiotemporally sequential delivery of antibacterial and anti-inflammatory drugs for the repair of MRSA-infected wounds" reported a series of ROS responsive HA-PBA/PVA (HPA) hydrogel, the hydrogel was prepared by phenylborate ester bond cross-linking between hyaluronic acid grafted 3-aminophenylboronic acid (HA-PBA) and polyvinyl alcohol (PVA) to achieve spatiotemporally controlled release of two kinds of drug to treat MRSA-infected wound. Overall, this manuscript has complete experiments and substantial data, but there are still some issues that need to be resolved before publication. The specific issues and suggestions are as follows:
1. What are the shortcomings of the hydrogels prepared in this manuscript that need to be improved to consider practical applications.

**Response:** Thanks for your comment. In practical applications, our dressings lack a removable effect. The hydrogel has good thermal stability between 25°C-40°C, so when applied to the skin surface, the hydrogel can still maintain its own stability even under body temperature conditions. However, in practice, the dressing change must not damage the wound, which requires the hydrogel to be removable, and we will improve this in subsequent studies. And we have supplemented these contents in the page 17 of the manuscript.

2. The format of references needs to be further reviewed, and there are still inconsistencies. Therefore, the author is requested to review the manuscript systematically again.

**Response:** Thank you for your suggestions. The format of the references has been modified. And these changes are indicated in the manuscript in red.
3. Figure 7c needs to be revised.

**Response:** Thanks for your comment. We have modified Figure 7c to make it consistent with the description in the article.

4. Why choose curcumin instead of other flavonoids? What are the advantages of curcumin?

**Response:** Thank you for your suggestions. The compounds of flavonoids have a wide range of biological activities such as antibacterial, anti-inflammatory and antioxidant [1]. The main reasons for selecting curcumin for this study are:

1. Curcumin is safe and has been widely used in the food and medical industries and is permitted as a food additive by the World Health Organization and the Food and Drug Administration of the United States of America, as well as by several countries [2].

2. Curcumin, with its polyphenolic structure and β-diketone functional group, is a more potent antioxidant inhibitor of lipid peroxidation than other flavonoids with a single phenolic hydroxyl group [3].

3. Studies on curcumin treatment of animal wounds have shown that endogenous production of transforming growth factor-β1 by curcumin during the wound healing phase may lead to increased granulation tissue deposition on the wound bed [4].

4. The industrial production of curcumin is more mature and the product is cheap and economical [5].

We've added the benefits of curcumin in the Introduction section of the manuscript in red.
5. How about the stability of the hydrogel? Especially thermal stability.

**Response:** Thanks for your comment. As shown in the figure, we tested the modulus change of the hydrogels in the range of 25°C-40°C, and found that the modulus of HPA2 and HPA2/PF hydrogels did not change much. In addition, we can clearly observe that the HPA2/PF hydrogel can still maintain the gel-forming state under the high temperature condition of 40℃. We provide an additional discussion of the thermal stability of hydrogels on page 17 of the manuscript and have placed the relevant figure in the SI, which is the Fig. S2. And these changes are in red.

6. How long does it take for the hydrogel to gelation?
**Response:** Thanks for your comment. Appropriate gelation time of the hydrogel is of much importance for the clinical or biomedical application. One milliliter of each sample (n = 3) was added into test tubes and shake with an oscillator at room temperature. The gel-forming state of the hydrogel was observed in time and the time was recorded.

| Hydrogels | Average gelation time (s) |
| --- | --- |
| HPA2 | 50.4 ± 2.4 |
| HPA2/PF | 69.3 ± 2.1 |

The average gelation time of the HPA2 hydrogel was 50.4 ± 2.4 S, while the gelation time of the HPA2/PF hydrogel was a little longer, about 69.3 ± 2.1 S, which may be due to the surfactant of Pluronic F127 [6]. However, the addition of Pluronic F127 made the modulus of the HPA2/PF hydrogel higher than that of the HPA2 hydrogel, which was due to the presence of hydrogen bonding between Pluronic F127 and the hydrogel. These changes are indicated in the page 13 of manuscript in red. And we have placed the relevant table in the SI, which is the Table S1.

**Reviewer: 2**
Comments to the Author
This manuscript describes the development of a ROS-responsive 3-aminophenylboronic acid/polyvinyl alcohol (HA-PBA/PVA) hydrogel that loaded with antibiotic moxifloxacin and curcumin-encapsulated pluronic F127 micelles as a material for treating MRSA-infected skin wounds. The release of moxifloxacin and curcumin was controlled sequentially. The hydrogel treatment resulted in faster wound closure and reduced inflammation in the MRSA-infected mouse skin wound model. This study is interesting. The manuscript is recommended for publication after minor revision.
1. In the abstract, the phrase “different hydrophobic and hydrophilic nature of moxifloxacin and curcumin” could be changed into “different hydrophilic and hydrophobic nature of moxifloxacin and curcumin”, since moxifloxacin is hydrophilic while curcumin is hydrophobic.

**Response:** Thanks for your comment. We've corrected the content in the abstract.

2. The Graphic Abstract could to be further optimized.

**Response:** Thank you for your suggestions. We've modified the Graphic Abstract in the article.

3. The text description below the mouse image in Figure 1e and 6a should be “MRSA infection or MRSA-infected wounds”.

**Response:** Thanks for your comment. We've changed that to “MRSA-infected wounds” in Figure 1e and 6a.
4. It is better to revise the texts and labels in Figure 1 and 2 to unify the format. In addition, the decrease and increase of IL-6 and TNF-a in Figure 1 is not very clear.

**Response:** Thank you for your suggestions. We have unified the text and label formatting in Figures 1 and 2 and labelled the decrease and increase of IL-1β and TNF-α clearly.

5. In Figure 3, such as Figures 3H and 3i, the lines seem to be a little blurred.
**Response:** Thanks for your comment. We've redrawn 3H and 3i in Figure 3 to make the lines contrast clearly.

**Reviewer: 3**
Comments to the Author
In this manuscript, the authors prepared ROS-responsive HA-PBA/PVA via hyaluronic acid-grafted 3-aminophenylboronic acid (HA-PBA) and phenylborate bond cross-linking between polyvinyl alcohol (PVA) (HPA) hydrogel achieves spatiotemporal controlled release of two active substances to treat MRSA-infected wounds. The authors verified the therapeutic effect of the hydrogel by establishing an infected mouse skin defect model. Overall, this is a good work for this journal. However, there are still some issues in this manuscript that require minor revised:
1. The manuscript lacks chemical characterization confirming hydrogel preparation.

**Response:** Thank you for your suggestions. Our analysis of the FT-IR spectrum was supplemented to the article as follows: “Meanwhile, as shown in Fig. 2b, the changes in the peaks at 1459 and 1517 cm^-1^ in the FT-IR spectrum came from benzene ring, and the peak at 1340 cm^-1^ is attributed to the stretching vibration of the B-O, once again proving the successful grafting of PBA.”

2. Figure 2c confirms the formation of Cur-PF micelles with a diameter of approximately 300 nm. What changes occur in the micelle size after curcumin is encapsulated?

**Response:** Thanks for your comment. Picture in Fig. 2c represents PF127 micelles encapsulated with curcumin, and TEM showed an average diameter of approximately 300 nm. And we also tested the diameter of Cur-PF micelles by using Dynamic Light Scattering (DLS), the experimental results show that it is distributed in the range of 220-458 nm, which is consistent with the TEM results in the article. The average diameter of Pluronic F127 micelles has been verified in the literature to be in the range of 68-70 nm [7]. Therefore, the diameter of the micelles becomes larger after loading curcumin. And these changes are indicated in the page 13 of the manuscript in red.

3. The hydrogel almost completely degraded within 12 days. Does this match the needs of the wound healing cycle?

**Response:** Thank you for your suggestions. The hydrogel HPA2/PF prepared in this study had 16.5% undegraded on day 12, which matches the healing ratio of MRSA-infected wounds. In our animal experiments, we can see that for MRSA-infected wounds our hydrogel has a very good therapeutic effect, with almost complete wound healing on day 14. Therefore, it is reasonable that 16.5% of the hydrogel was still present on day 12. In practice, if it is really necessary, we will also change the dressings according to the condition of the wound.
4. Figures 3h and 3i should indicate the concentration of H2O2 used.

**Response:** Thanks for your comment. In the section of ROS responsive drug release of hydrogels and Figures 3h and 3i, we added the concentration of H_2_O_2_.

5. The novelty of the manuscript needs to be clarified.

**Response:** Thank you for your suggestions. The novelty of our manuscript is the spatiotemporal delivery of two different drugs. This is stated in the fourth paragraph of the Introduction section of our manuscript. For MRSA-infected wounds, timely treatment is needed. The use of antibiotics can easily lead to drug resistance, so medical materials with drug delivery properties are of great advantage for the treatment of MRSA-infected wounds. Most of the current hydrogels for the treatment of MRSA-infected wounds deliver only one drug, and for wounds, the treatment needs are different at different stages. Firstly, after the wound is formed, bacteria colonize, which in turn causes inflammation. Therefore, our hydrogel dressing, upon contact with MRSA-infected wounds, responsively first rapidly releases moxifloxacin for sterilization, and then slowly releases curcumin to regulate wound inflammation, thus achieving the purpose of wound repair.
6. How the spatiotemporal delivery of two drugs is achieved during the treatment of infected wounds?

**Response:** Thanks for your comment. When HPA/M&Cur-PF hydrogels are applied to MRSA-infected wounds, excessive ROS accumulated in the wound respond to the phenylborate bonds in the hydrogel network. Due to the spatial difference caused by the different forms of moxifloxacin and curcumin present in the hydrogel, when ROS responds to the phenylborate bond of the hydrogel, the hydrogel network dissociates, and moxifloxacin (M) and curcumin encapsulated in the PF micelles (Cur-PF) are released to the wound site, at which point the two drugs are delivered to the wound site still with spatial difference. After hydrogel disintegration, the antimicrobial drug moxifloxacin is released into the wound site and can act immediately to rapidly sterilise the wound. However, Cur-PF has a slow-release effect on curcumin, so curcumin can be gradually released into the wound site by the slow-release effect of the micelles, which results in the temporal variability of the two drugs. Regarding the differences in the release of moxifloxacin and curcumin, this can be verified in the ROS responsive drug release of hydrogels section of the article. These are mentioned on pages 4-6 of the manuscript.

For MRSA-infected wounds, the use of both drugs in a temporally sequential delivery, first allowing the antimicrobial drug to act to kill the bacteria at the wound site, and then allowing the anti-inflammatory drug to act as a sustained slow anti-inflammatory, is undoubtedly a good therapeutic strategy.

**Reviewer: 4**
Comments to the Author
1. What is the reason why the author finally chose PVA with concentration of 1, 2 and 3 wt% to prepare hydrogel?

**Response:** Thank you for your suggestions. In the pre-experiment, we set up a PVA concentration gradient of 1, 1.5, 2, 2.5, and 3 wt% PVA concentration to investigate the adhesion force of HPA hydrogels. It was found that the adhesion of the hydrogels showed a tendency to increase and then decrease with the increase of PVA concentration and the highest adhesion of 4 KPa was observed at 2 wt% PVA concentration when the cohesion of the hydrogels was the strongest. The difference in adhesion of the hydrogels with PVA concentrations of 1 and 1.5 wt% and 2.5 and 3 wt% PVA concentrations was not significant, so the final selection of 1, 2 and 3 wt% PVA concentrations was chosen.

2. This article lacks the characterization of Cur-PF micelle.

**Response:** Thanks for your comment. As shown in the figure, we tested the particle diameter of Cur-PF using Dynamic Light Scattering (DLS), and the experimental results show that it is distributed in the range of 220-458 nm, which is consistent with the TEM results in the article. These contents were also added in the manuscript on the page 13 in red, and we have placed the relevant figure in the SI, which is the Fig. S1.
3. Spatiotemporally sequential delivery of antibacterial and anti-inflammatory drugs is the highlight of this work, please explain why there is no experiment demonstrating the spatiotemporal sequential delivery of two drugs appearing in the full manuscript.

**Response:** Thank you for your suggestions. The difference in spatial location of the presence of the two drugs is clear in the preparation of the hydrogels, where moxifloxacin is directly involved in the hydrogel network, while curcumin is encapsulated in the PF micelles. The drug release test in the article was to verify the temporal release difference of the two drugs, and the temporal release difference indirectly reflects their spatial difference. When excess ROS responds to the phenylborate bond in the hydrogel, the hydrogel network disintegrates and moxifloxacin is released directly, but curcumin is released slowly because it is encapsulated in the PF micelles.
4. The graphic abstract of this work needs to be re-conceived, and the existing graphic abstract does not show the highlight of this article: spatiotemporally sequential delivery of antibacterial and anti-inflammatory drugs.
**Response:** Thanks for your comment. We've redrawn the Graphic Abstract in the article.

**Reviewer: 5**
Comments to the Author
In this manuscript, the authors prepared a kind of ROS responsive HPA hydrogel to achieve spatiotemporally controlled release of two kinds of drug to treat MRSA-infected wound. The manuscript structure is complete and the data is sufficient, so I think this paper is suitable to be published in Regenerative Biomaterials. Here are some questions for the authors’ consideration.
(1) The Author should calculate out the grafting rate of HA-PBA based on the 1H-NMR in figure 2a.

**Response:** Thank you for your suggestions. The chemical shifts (*δ*, ppm) of the peaks were assigned as below: 7.58 (m, 4H, A), 1.89 (s, 3H, B). It could be seen that phenylboronic acid was successfully grafted. Through integral calculation, the grafting ratio of PBA was 14.2%. And these contents were also added in the manuscript in red.
(2) The author should provide the rheological frequency sweep data for HPA hydrogel to prove the reversible physical crosslinking property.

**Response:** Thanks for your comment. As shown in the figure, shear rate scanning measurements indicate that all hydrogels in this experiment exhibit shear-thinning behavior, where the viscosity of the material depends on the shear force, and thus the hydrogels we prepared are injectable. These contents were also added in the manuscript in red, and we have placed the relevant figure in the SI, which is the Fig. S3.
(3) The author also should use rheological time sweep data to further prove the ROS responsive property of HPA hydrogel.

**Response:** Thank you for your suggestions. As shown in the figure, the modulus of HPA2 hydrogels and HPA2/PF hydrogels were tested under 1 mM H_2_O_2_ or PBS for the same time, respectively. At 10 min, the modulus of the hydrogel in PBS did not change much compared to the initial value, but the modulus of the hydrogel in H_2_O_2_ decreased dramatically by about 200 Pa. At 20 min, the modulus of the hydrogel in PBS decreased due to the swelling and absorbing of water, and at this time, the hydrogel in H_2_O_2_ was close to the state of non-gelation. These contents were also added in the manuscript in red, and we have placed the relevant figure in the SI, which is the Fig. S4.
(4) What’s the control group for antibacterial test in figure 4? The author should give detailed description.

**Response:** Thanks for your comment. The control group in Figure 4 is just the holes punched on the agar plate by the punch, so the diameter size and shape of the control group do not change with time and environmental factors, and at the same time to ensure that the initial diameter is the same between every group, and to confirm whether the experimental group formed a ring of inhibition around the holes. These contents were also added in the manuscript in red.
(5) Maybe the author can add the HPA2 hydrogel group to the antibacterial test to further clarify the impact of hydrogel network on the antibacterial property of HPA2/M/Cur-PF hydrogel.
**Response:** Thank you for your suggestions. In the inhibition zone test, we have already done a study on the antibacterial effect of HPA2 hydrogel group, and the results of the test show that it does not have antibacterial effect. This is mainly due to the fact that the components of the HPA2 hydrogel group, hyaluronic acid, 3-aminobenzeneboronic acid and polyvinyl alcohol, do not have antibacterial effects by themselves.

**Reviewer: 6**
Comments to the Author
This manuscript entitled " ROS-responsive hydrogels with spatiotemporally sequential delivery of antibacterial and anti-inflammatory drugs for the repair of MRSA-infected wounds" reports a hydrogel for infected wound treatment based on hyaluronic acid, phenylboronic acid, polyvinyl alcohol, curcumin, and moxifloxacin. Curcumin and moxifloxacin were loaded into the hydrogel in different ways to achieve different spatio-temporal sequential delivery of the two drugs. In addition, the hydrogel exhibited degradation properties, self-healing properties, antimicrobial properties, anti-inflammatory properties and biocompatibility properties. Furthermore, the hydrogel was effective in promoting closure of infected wounds and reducing inflammation and increasing collagen deposition. However, I suggest that the author modify the following questions before publishing it in the journal.
1. In this manuscript, the authors mention several times that hydrogels have spatio-temporal sequential delivery properties. The authors are requested to further explain the specific manifestation of the spatio-temporal sequential delivery property.

**Response:** Thanks for your comment. When HPA/M&Cur-PF hydrogels are applied to MRSA-infected wounds, excessive ROS accumulated in the wound respond to the phenylborate bonds in the hydrogel network. Due to the spatial difference caused by the different forms of moxifloxacin and curcumin present in the hydrogel, when ROS responds to the phenylborate bond of the hydrogel, the hydrogel network dissociates, and moxifloxacin (M) and curcumin encapsulated in the PF micelles (Cur-PF) are released to the wound site, at which point the two drugs are delivered to the wound site still with spatial difference. The antimicrobial drug moxifloxacin is released into the wound site and can act immediately to rapidly sterilize the wound. However, Cur-PF has a slow-release effect on curcumin, so curcumin can be gradually released into the wound site by the slow-release effect of the micelles, which results in the temporal variability of the two drugs. For MRSA-infected wounds, the use of both drugs in a temporally sequential delivery, first allowing the antimicrobial drug to act to kill the bacteria at the wound site, and then allowing the anti-inflammatory drug to act as a sustained slow anti-inflammatory, is undoubtedly a good therapeutic strategy.

2. In the manuscript, how the authors determined the concentrations of curcumin and moxifloxacin.

**Response:** Thank you for your suggestions. In this study, the concentration of both curcumin and moxifloxacin in the hydrogel was 1 mg/mL.

It has been shown that curcumin has an effect on wound healing promotion from an anti-inflammatory point of view [8]. And we need to clarify the anti-inflammatory concentration of curcumin, so different concentrations of curcumin micellar hydrogel leachate were used in the LPS-induced macrophage assay, and finally determined that 1 mg/mL curcumin in the hydrogel has an anti-inflammatory effect.

In the case of moxifloxacin, we referred to drug concentrations in wound dressings from previous studies [9]. When the moxifloxacin concentration was 1 mg/ml, the hydrogel showed good bacterial inhibition in the circle of inhibition test.
3. In the manuscript, the analysis for 1H NMR of HA-PBA was too simple.

**Response:** Thank you for your suggestions. We have enriched the relevant content as follows “The chemical shifts (*δ*, ppm) of the peaks were assigned as below: 7.58 (m, 4H, A), 1.89 (s, 3H, B). It could be seen that phenylboronic acid was successfully grafted. Through integral calculation, the grafting ratio of PBA was 14.2%.”
4. In the manuscript, there was significant cytotoxicity in the HPA 2/M&Cur-PF group, but the authors describe it as not significantly different from the control group and request the authors to verify the data.

**Response:** Thanks for your comment. The difference in cell viability between the HPA2/M&Cur-PF hydrogel group and the control group were incorrectly stated in our previous article and has been revised as follows: "The cell viability of the HPA2/M&Cur-PF group compared to the control group was 80.3%, 82.1%, and 94.4% in the first three days, respectively. Although the cell viability of the experimental group was slightly decreased in the first two days of testing compared to the control group, the significant differences were all P<0.05, indicating that the material was not toxic."

**References**

1.	Maheshwari RK, Singh AK, GaddipatiJ et al. Multiple biological activities of curcumin: a short review. Life sci 2006;**78**:2081-2087.

2.	Ali A, Jaber N. Pharmacological aspects of curcumin: Review article. Int J Pharmacogn 2018;**5**:313-26.

3.	Indira PriyadarsiniK. Chemical and structural features influencing the biological activity of curcumin. Curr Pharm Des 2013;**19**:2093-2100.

4.	Sidhu GS, Mani H, Gaddipati JP et al. Curcumin enhances wound healing in streptozotocin induced diabetic rats and genetically diabetic mice. Wound Repair Regen 1999;**7**:362-374.

5.	Yixuan L, Qaria MA, Sivasamy S et al. Curcumin production and bioavailability: A comprehensive review of curcumin extraction, synthesis, biotransformation and delivery systems. Ind Crops Prod 2021;**172**:114050.

6.	Antunes FE, Gentile L, Rossi CO et al. Gels of Pluronic F127 and nonionic surfactants from rheological characterization to controlled drug permeation. Colloids Surf B 2011;**87**:42-48.

7.	Basak R, Bandyopadhyay R. Encapsulation of hydrophobic drugs in Pluronic F127 micelles: effects of drug hydrophobicity, solution temperature, and pH. Langmuir 2013;**29**:4350-4356.

8.	Shehzad A, Rehman G, Lee YS. Curcumin in inflammatory diseases. Biofactors 2013;**39**:69-77.

9.	Garms BC, Borges FA, de Barros NR, Marcelino MY, Leite MN, Del Arco MC, de Souza Salvador SL, Pegorin GSA, Oliveira KSM, Frade MAC. Novel polymeric dressing to the treatment of infected chronic wound. Appl Microbiol Biotechnol 2019;**103**:4767-4778.
